# Role of the Ghrelin System in Colorectal Cancer

**DOI:** 10.3390/ijms23105380

**Published:** 2022-05-11

**Authors:** Aldona Kasprzak

**Affiliations:** Department of Histology and Embryology, Poznan University of Medical Sciences, Święcicki Street 6, 60-781 Poznan, Poland; akasprza@ump.edu.pl; Tel.: +48-61-8546441

**Keywords:** ghrelin system, colorectal cancer, prognostic factors, colorectal cancer-associated obesity, ghrelin, analogue therapy

## Abstract

The ghrelin system contains several components (e.g., ghrelin with growing number of alternative peptides, growth hormone secretagogue receptors (GHS-Rs), and ghrelin-O-acyl-transferase (GOAT) and participates in regulation of a number of key processes of gastrointestinal (GI) tract cancer progression, including cell proliferation, migration, invasion, apoptosis, inflammation, and angiogenesis. However, its exact role in promoting or inhibiting cancer progression is still unclear. Colorectal cancer (CRC) is one of the most common human malignancies worldwide. Molecular studies suggest an autocrine/paracrine mechanism for the secretion of ghrelin in colorectal carcinogenesis and its contribution to its initial stages. However, the signalling pathways of CRC development involving the ghrelin system are poorly understood. Potential mechanisms of colon carcinogenesis involving components of the ghrelin system were previously described in an animal model and in in vitro studies. However, the diagnostic–prognostic role of serum ghrelin concentrations, tissue expression, or genetic changes of this system in various stages of CRC progression remains an open case. Thus, the aim of this study is to discuss the role of the ghrelin system in colon carcinogenesis, diagnostics and CRC prognostics, as well as the results of studies on the use of ghrelin and its analogues in the therapy of CRC-related syndromes (e.g., cachexia and sarcopenia).

## 1. Introduction

**Figure 1:**
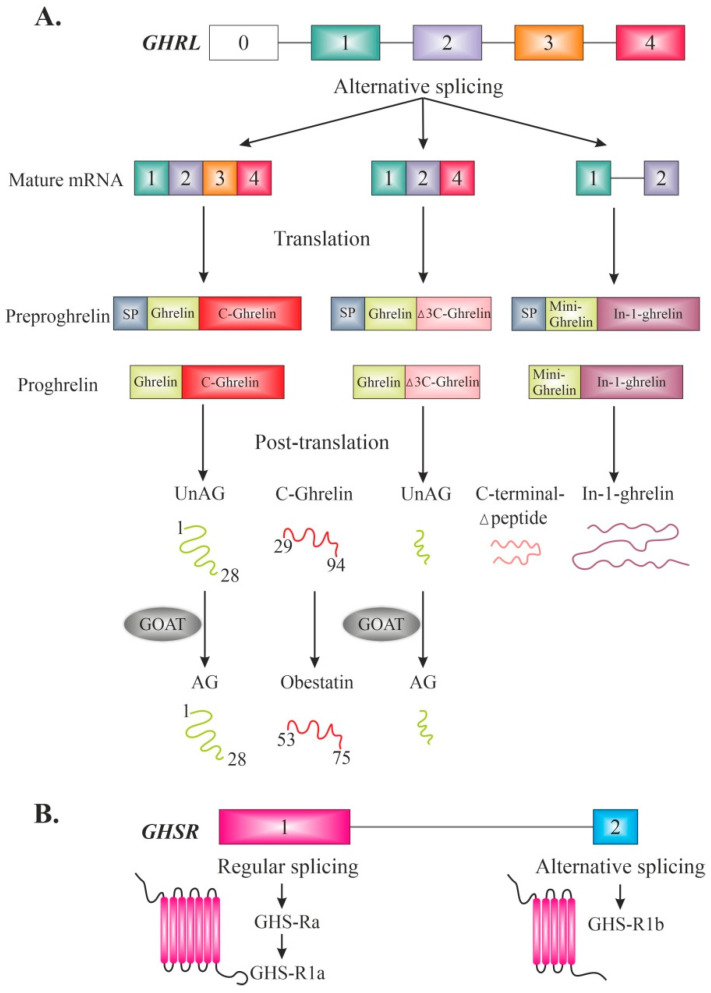
Schematic diagram of the genes encoding human ghrelin (*GHRL*) (**A**) and the ghrelin receptor (*GHSR*) (**B**). The functionally relevant *GHRL* and *GHRS* gene-derived transcripts and the most important peptides are specified. Exons are marked as boxes, and introns as lines. [AG-acylated ghrelin; GOAT-ghrelin-O-acyltransferase; SP-Signal peptide; UnAG-unacylated ghrelin].

The components of the ghrelin system comprise a complex family of peptides, controlling multiple pathophysiological processes. The system includes acylated ghrelin (AG), des-acyl (or unacylated ghrelin, UnAG), and a growing number of alternative peptides (e.g., obestatin, C-terminal Δpeptide, and In-1 ghrelin), growth hormone secretagogue receptors (GHS-Rs), and ghrelin-O-acyltransferase (GOAT) [1,2,3,4,5,6,7] (Figure 1A). These diverse transcripts and proteins are encoded by the human ghrelin gene (*GHRL*) located on the short arm of chromosome 3 [2,6,8,9]. The main product of *GHRL* is a 28-amino acid (aa) peptide, simply called ghrelin, which is a natural endogenous ligand for pituitary GHS-R, and a potent stimulator of growth hormone (GH) release [10,11,12,13]. Subsequent studies on the biology of ghrelin revealed it to be a multifunctional hormone, responsible for hypothalamic regulation of energy homeostasis, as a meal initiator, and many other physiological effects [1,9,14,15,16]. However, it should be emphasised that the old term “the hunger hormone” does not adequately capture the wide range of roles that are now attributed to ghrelin [17].

The GHS-R is an orphan G protein-coupled receptor, distinct from the receptor for the GH-releasing hormone [18,19] and is now formally known as the ghrelin receptor with high constitutive activity [20,21]. In humans, it is encoded by a conserved single-copy gene (*GHSR*) located on chromosome 3 [22]. Expression of this gene generates two mRNA species named GHS-R1a and GHS-R1b [21,23,24] (Figure 1B).

GHS-R strongly binds AG (poorly responsive to UnAG) and is generally well conserved across species [21,25]. Ghrelin is activated through peptide acetylation, catalysed by GOAT, a membrane-bound enzyme that attaches eight-carbon octanoate to a serine residue in ghrelin and thereby acylates inactive ghrelin to produce active ghrelin (AG) [7,26]. Only this active form has the ability to bind GHS-R1a, and is responsible for its GH-releasing capacity, and most likely other endocrine actions [7,10,27,28]. Ghrelin acetylation is a necessary condition to cross the blood–brain barrier [29]. The acetylation process itself takes place in the human liver [30]. AG makes up ~10% of total plasma ghrelin, and is responsible for appetite-stimulation, hunger signalling and other metabolic effects [7,9]. In turn, while UnAG does not evoke orexigenic effects, it is directly involved in muscle tissue metabolism (reviewed in: [9]).

While circulating ghrelin is secreted by the X/A-like enteroendocrine cells (EECs) of the oxyntic (parietal) mucosa of the gastric fundus, tissue expression of ghrelin and GHS-R1a was identified in most other central and peripheral tissues [19,31,32]. In stomach oxyntic gland cells, ghrelin is subject to co-expression with other peptides, e.g., nesfatin-1 [16,33,34], and period circadian regulator 1 (PER1) and PER2 proteins [35].

Circulating ghrelin concentration varies during the circadian cycle. Healthy individuals exhibit initial nocturnal elevation, declining towards the morning. Furthermore, ghrelin concentration increases before meals and decreases after consumption [10,36,37,38]. Ghrelin acts in a secondary peripheral circadian clock (or non-suprachiasmatic nucleus), together with melatonin, GH, insulin, adiponectin, playing an important role in the maintenance of the circadian rhythm in the brain and peripheral organs [39]. Interestingly, *per1* and *per2* deletion causes cession of rhythmic ghrelin expression [35], which might lead to a range of clinical consequences (reviewed in: [40]).

The ghrelin system serves a number of physiological functions in the gastrointestinal (GI) tract, e.g., the regulation of motility, protection of mucosal tissue, secretion of gastro-pancreatic peptides, microbiome homeostasis and inflammation in aging [15,25,41,42,43,44]. It also plays an important role in the pathogenesis of a range of diseases, including functional GI tract disorders [45,46], inflammatory bowel diseases (IBD), coeliac disease, infectious diseases, and diabetic gastroenteropathy [47,48,49,50].

An increasing number of reports indicate the participation of ghrelin in the regulation of a range of tumour-related processes, including tumour metastasis [51]. The components of the ghrelin system are expressed in tissues and cell lines from GI tract cancers, including human neuroendocrine tumours [4,52,53,54], GI stromal tumours (GIST) [55,56], oesophageal, gastric, pancreatic and liver cancers [57,58], and colorectal cancer (CRC) [58,59,60,61,62].

Colorectal cancer is one of the most common human cancers, both in terms of incidence and morbidity [63,64]. There is a number of hereditary/familiar and lifestyles factors playing important roles in the pathogenesis and progression of this heterogeneous tumour [65,66,67,68,69]. Nonetheless, the search for new biomarkers, crucial in early diagnostics of this cancer, continues [70,71]. Potential mechanisms of colon carcinogenesis involving components of the ghrelin system were previously described in an animal model and in in vitro studies [51,58,72,73,74]. However, the diagnostic-prognostic role of serum ghrelin concentrations, tissue expression, or genetic changes of this system in a various stages of CRC progression remains an open case. Thus, the aim of this study is to discuss the role of ghrelin signalling in colon carcinogenesis, diagnostics and CRC prognostics, and the potential use of ghrelin and its analogues in therapy of CRC-related syndromes (e.g., cachexia and sarcopenia).

The first part of this review discusses the role of the ghrelin system in colon physiology. Furthermore, the potential participation of the ghrelin system in CRC-associated obesity, a common CRC risk factor, will be highlighted. The next part will concern the role of the components of the ghrelin system in colon carcinogenesis based on a review of the findings in patients with CRC, in animal models, as well as in in vitro studies.

## 2. Roles of the Ghrelin System in the Intestine

### 2.1. Cellular Sources of Ghrelin in the Normal Large Intestine

The presence of ghrelin-producing endocrine cells, as well as GHS-R, in the GI tract wall, from the stomach to the colon, was described in rats and humans more than 20 years ago. A notably lower number of ghrelin-positive cells can be observed in the large intestine, in contrast to oxyntic glands in the fundus of the stomach and upper intestinal regions [19,75]. The abundance of ghrelin-positive cells in the stomach mucosa is evolutionarily conserved between mammals and lower chordates [76]. In rat stomach, the amount of UnAG is higher than that of AG [12,77]. While obestatin has been detected in rat stomach endocrine cells and within the myenteric plexus [12], in the human gastric fundus its amount is relatively low, compared to ghrelin [78].

Ghrelin was detected in basal cytoplasm of so-called X/A-like cells, making up around 20% of chromogranin A-immunoreactive EECs in human gastric fundus. The cells were round or elliptical, closed-type, with strong electron-dense granules, 120 ± 30 nm in size [49,75,79]. In rodents, X/A cells are analogous to human P/D1 cells, in which round, electron-dense granules are slightly bigger, around 147 ± 30 nm in size. Further rat GI tract studies demonstrated ghrelin expression in lumen-contacted opened-type cells, the number of which increases between the stomach and the lower GI tract. Using electron microscopy, UnAG were localised mainly in the perinuclear area, while AG was present in the periphery of the cytoplasm. In rat large intestine, two histologically distinct types of ghrelin-producing cells were identified: opened-type, mainly regulated by luminal signals, and closed-type, regulated by other hormones, neural stimulation or mechanical distension [80,81].

Due to the development of intestinal hormone-producing cell visualisation techniques, 4 EEC lineages were identified in the mouse GI tract, including ghrelin/motilin (or M) cells. In most cases, co-expression of ghrelin and motilin was observed, particularly in the small intestine. However, no such co-expression was noted in the case of other investigated intestinal hormones [82]. Other authors demonstrated that, beside M cells, ghrelin is also present in other cells around the entire GI tract, e.g., EC, S, I, L cells (all cholecystokinin (CCK)-producing cells), co-localizing with other hormones, e.g., 5-hydroxytryptamine (HT-5), secretin, glucose-dependent insulinotropic peptide (GIP), glucagon-like peptide 1 (GLP-1), neurotensin, and PYY [79,83]. In studies on human large intestine EEC populations (normal sigmoid colon) the presence of ghrelin mRNA, or any ghrelin-positive cells was not detected among the four most expressed hormones: 5-HT, peptide YY (PYY), GLP-1 and somatostatin (SST) [84]. Ghrelin expression has also been shown to be present in human pancreas, β-cells [85,86], α-cells [87], and epsilon cells [88].

Ghrelin-producing cells can already be detected in early stages of stomach, intestine, pancreas and lung development in humans, rats, and dogs [49,89,90,91]. In the stomach, such cells were present in the 11th week of pregnancy, most numerously during the second trimester (~34%), and in infants (~28%) [90]. In the duodenum, similarly to the stomach, chromogranin A- and ghrelin-positive cells were identified in the 10th and 11th week of pregnancy, respectively. The first trimester of prenatal development was characterised by the presence of the highest number of ghrelin-positive endocrine cells in the duodenum. Their number was progressively decreasing, to increase again during the early postnatal period, compared to the second trimester of development [91].

Subsequent studies have also shown a wide distribution of both ghrelin receptors (GHS-R1a and GHS-R1b) in various normal human organs. In the context of the GI tract, GHS-Rs expression was detected in the stomach, intestines and large glands (liver, pancreas) [20,85,92,93]. Both human and rat stomach and colon showed expression of GHS-Rs in neurons and their protrusions. Receptor expression was also detected in cells associated with gastric glands, EECs, and/or mast cells. Smooth muscle and epithelial cells were devoid of this immunoreactivity and only rats showed GHS-R expression on nerve fibres associated with muscle layers [94].

In the human foetus, the expression of the active form of GSH-R1a was already detected in the 10th week of gestation in endocrine cells of the stomach antrum and corpus, as well as in the duodenal epithelial cells in the 11th week of gestation. GHS-R1b was detected in the second trimester of gestation (16th week) in epithelial cells of duodenum and in the longitudinal muscle layer of the antrum and corpus of the stomach [91].

Regarding normal human large intestinal tissue analysis using RT-PCR, expression of ghrelin mRNA was lowered between the left and right colon, while the levels of GHS-R1b showed an opposite relation. In turn, colon tissues did not demonstrate production of GHS-R1a mRNA [93]. Furthermore, in research based on immunocytochemistry (IHC) (with polyclonal antibodies against GHS-R1a), expression of this receptor was detected in ~22% CRC-neighbouring normal colorectal epithelial cells analysed on tissue microarray (TMA) slides. However, the mentioned publication did not investigate the expression of ghrelin and GHS-R1b [95]. Nonetheless, later studies confirmed ghrelin [59,61,96], GHS-R1a [59,61] and GHS-R1b [59] immunoreactivity in normal colorectal tissue samples. While most recent reports also demonstrated GHS-R expression, the polyclonal antibodies on which they were based did not determine the GHS-R subtype. Cytoplasmic IHC reaction to the “general” GHS-R mostly concerned cells of normal large intestine tissue [96], confirming the previously reported cellular localisation of both GSH-R1a and GSH-1b subtypes [59,61,95]. Ghrelin immunostaining was more differentiated than GHS-Rs, from a mixed (nuclear-cytoplasmic) [59] to a solely cytoplasmic pattern [61,96]. In normal human large intestine, production of GOAT was also demonstrated, with its majority in the right colon vs. the left colon [97].

Recent studies based on the qPCR method confirm that ghrelin and GHS-R1a are present in a large proportion of normal tissues (in more than 80%) of healthy controls, higher than that of In-1-ghrelin and GHS-R1b (about 40% of the samples). The least abundant expression was demonstrated in the case of the GOAT protein (in less than 20% of samples). Unfortunately, the authors do not state from which exact fragments of the intestine the normal tissues were taken, describing them generally as normal control tissues from healthy donors (n = 14) [54]. Recently, the presence of GOAT mRNA was also demonstrated in the human liver [30]. GOAT, similarly to other components of the ghrelin system, is also commonly localised in the GI tract, involving not only the rodent stomach, but also the pancreas, small intestine, and colon [98,99]. While in humans it is mostly present in the intestine, GOAT mRNA expression was also observed in other tissues/organs, such as liver, stomach, pancreas, skeletal muscle, heart, bones [97], and plasma [100].

In summary, studies indicate that ghrelin/GHS-Rs expression is very common in normal human organs and tissues. Expression of ghrelin occurs most abundantly in the stomach, often in co-localization with other hormones. In the colon, ghrelin is produced by two types of cells: opened- and closed-type cells. In turn, expression of GHS-Rs occurs in both the stomach, intestines (including colon), and large GI tract glands (liver, pancreas). Immunoreactivity to both receptor types (GHS-R1a and GHS-R1b) was also confirmed in normal colorectal tissue samples.

### 2.2. Effects of Ghrelin on the GI Tract in Physiology

The actions of ghrelin and other peptides of their family result from their neurohormonal, paracrine, and autocrine activity [13,94,101]. Its broad regulatory and metabolic effects are related to its production outside the hypothalamus, and the presence of its receptors in numerous organs and human tissues. A number of factors, including nutrients, play a major role in the modulation of ghrelin action at a central level [9].

In relation to the GI tract, the main activities of the ghrelin/GHS-Rs comprise gastric acid secretion and motility, alteration of appetite and maintenance of energy balance [20,41,43,102,103]. Both ghrelin and motilin can stimulate stomach emptying [43]. Active ghrelin (AG), UnAG, and nesfatin-1 were described as the main regulators of food intake and body weight [16,33].

The latest mouse model research suggests that the role of AG in increasing food intake and body weight are reliant on direct activation of GHS-Rs expressed on somatotrophs, while its glucoregulatory actions are independent of GHS-R expression by these cells [104]. Promotion of eating behaviour, stimulation of gastric motility and hydrochloric acid secretion also occurs through the nitric oxide (NO) pathway. Ghrelin protects the gastric mucosa through stimulation of blood flow and NO-mediated hyperaemia. Carbon monoxide (CO) is also involved in ghrelin-induced gastroprotection (reviewed in: [105]).

Twenty years ago, it was already shown in rodents that ghrelin exerts significant effects on GI tract function, both through the enteric nervous system (ENS) [94], as well as vagus nerve-dependent mechanisms [106]. Soon after its discovery, it was recognised as a prokinetic agent in the stomach, due to its homology to motilin [42,107]. Thus, it was shown to exhibit gastroprokinetic and strong orexigenic activity, by acting on hypothalamic neuropeptide Y (NPY) and the Y(1) receptor, which disappeared after vagotomy. Ghrelin decreased gastric afferent transmission, in contrast to anorexigenic peptides, which usually increase this activity [106]. The ENS and vagus nerve-dependent actions of this protein may complement and reinforce each other and/or have distinct roles and functions [94]. In humans, the mechanisms increasing gastric motility, in contrast to rodents, probably do not depend on stimulation of enteric motor neuron activity [108].

It has been suggested that ghrelin (together with motilin) is involved in the generation of migrating myoelectric (motor) complex (MMC) activity and, together with other peptides (e.g., gastrin, CCK, serotonin), in the generation of slow wave spikes, resulting in peristaltic or segmental contractions in various sections of the small intestine and colon [40]. In humans, secretion of endogenous ghrelin and UnAG, unlike motilin, is not associated with MMC activity [109]. Administration of exogenous ghrelin, however, initiates gastric phase III MMC activity in humans that is not mediated by motilin release. This is accompanied by prolonged increased tension of the proximal part of the stomach [110].

Recent studies have shown that ghrelin produces a biphasic effect on food intake, indirectly affecting energy expenditure and nutrient distribution. This effect requires the integrity of Agouti/NPY peptide-producing neurons in the arcuate nucleus of the hypothalamus. Furthermore, it has been described that various autonomic, hormonal, and metabolic satiety signals transiently counteract ghrelin-induced food intake [111].

In addition to increasing gastric motility, ghrelin also affects gastric hydrochloric acid secretion [41,102], with the mechanisms of this activity remaining a subject of discussion [15,25]. Intravenous administration of this peptide also stimulated gastrin secretion [112], while its enteral intake increased CCK and pancreatic enzyme secretion [113]. In humans, ghrelin has also been shown to stimulate the release of SST and pancreatic polypeptide (PP) [114].

In turn, the effect of the ghrelin system on insulin secretion by the pancreas is more controversial. Some of the authors describe its inhibiting effect on glucose-stimulated insulin release from human and rodent models of diabetes mellitus [115], while others implicate it in basal or glucose-induced insulin release in humans [116]. In turn, other reports describe identical fasting-induced serum levels of leptin and insulin in wild-type and GHS-R-null mice [37]. There are also results indicating an increase of insulin levels in experimental diabetes [117], both in diabetic rats and the CRL110065 beta cell line, caused by both forms of ghrelin [86]. Similarly to AG, both UnAG and obestatin counteracted streptozotocin-induced high glucose levels and improved plasma and pancreatic insulin levels, which were lowered by this diabetogenic compound [117]. Stimulation of gastrin and insulin secretion after intravenous administration of ghrelin in rats was observed by Lee et al. [112] Furthermore, a blockade of pancreatic ghrelin secretion significantly increased glucose-induced insulin release. This indicates that pancreatic islet-derived ghrelin physiologically reduces insulin release in rodents by directly inhibiting β cells and promoting SST secretion from δ cells (via paracrine and autocrine pathways) (reviewed in: [101]).

In colon, a ghrelin-mediated prokinetic effect has been demonstrated in fish and selected birds, but not in rodents and humans [94,118]. The possible reasons for the lack of such an effect on human colon motility are still discussed [42]. It has also been observed that colonic motility is activated by ghrelin only when it is administered centrally through an injection into the medial hypothalamic nucleus [119]. Nonetheless, the resulting contraction is less intense than that initiated by the lumbosacral plexus [120,121]. Moreover, activation of GHS-Rs in the lumbosacral spinal cord has been shown to trigger coordinated propulsive contractions that empty the colon [120]. Additionally, it has been observed that acylation of ghrelin is necessary to promote such contractions, and UnAG counteracts this effect [121].

### 2.3. Regulation of Ghrelin Secretion in the GI Tract

The main physiological role of ghrelin is to promote an increase in food intake [16,25,106,122]. Circulating ghrelin shows a diurnal pattern with a preprandial rise, postprandial fall and a maximum peak at 02:00. Furthermore, ghrelin secretion is reduced by positive energy balance [36]. Consumption of drinks containing carbohydrates and protein results in a significantly greater decrease in ghrelin concentrations compared to lipid drinks [123]. A lower number of ghrelin-positive cells was also observed during *Helicobacter pylori* infection in the stomach, with a subsequent increase after eradication of the infection [124]. Moreover, diets and dietary-induced weight loss have been linked with an increase of ghrelin serum levels [122]. Plasma GOAT shows a negative correlation with ghrelin and a positive correlation with BMI. As GOAT is the only enzyme that acylates ghrelin, and ghrelin is the only substrate for GOAT in the human proteome; it ultimately contributes to the development or maintenance of anorexia and obesity [7,100]. The release of ghrelin from the stomach is also inhibited by L-cysteine, which acts as an H_2_S donor [105]. The ghrelin opposite strand/antisense non-coding RNA (GHRLOS), through an overlapping genomic arrangement with *GHRL*, is also involved in the regulation of ghrelin signalling [13,125].

Ghrelin-releasing endocrine cells are stimulated by the sympathetic nervous system through β-adrenoceptors, or by vagus via muscarinic receptors. In turn, the sympathetic nervous system is a ghrelin secretion inhibiting factor, acting via α-adrenoceptors [126].

Local ghrelin secretion is also regulated by different GI tract peptides/cytokines/hormones. A stimulating effect is evoked by, e.g., adrenaline, noradrenaline, endothelin 1 and -3, secretin, nesfatin-1, endocannabinoids, and glucagon, while inhibition results from the action of, e.g., SST, GRP, GLP-1, CKK, PYY, bombesin, insulin, leptin and interleukin 1β (IL-1β) [33,34,106,127].

## 3. The Ghrelin System in CRC-Associated Obesity

Ghrelin is an important physiological regulator of lipid metabolism (both adipogenesis and lipogenesis). In addition to obesity, insulin resistance, type 2 diabetes mellitus (T2D) and metabolic syndrome (MetS) are also associated with a paradoxical decrease in circulating ghrelin levels. However, these pathologies are associated with dramatic decreases in UnAG concentrations, while plasma AG concentrations remain unchanged or increase (reviewed in: [128]).

Many GI tract malignancies are associated with obesity (including CRC), defined specifically by increased body mass index, most likely due to environmental rather than genetic factors [129]. The pathogenesis of obesity is related to a specific metabolic condition characterised by hyperinsulinemia or insulin resistance, as well as increased levels of leptin, IGF-1, and/or serum free fatty acids levels [67,129,130].

Although the underlying molecular mechanisms are still poorly understood, the role of obesity-related adipokines in pathogenesis of CRC-associated obesity is often highlighted [130,131]. Ghrelin was among the most frequently mentioned cytokines produced by adipocytes, along with adiponectin, leptin, and resistin. Leptin appears to play an especially important role in ghrelin regulation [67,129,130]. The course of obesity is characterised by changes in adipose tissue (AT)-secreted adipokine levels, including an increase in local ghrelin secretion. All these changes gave rise to the hypothesis that unfavourable adipokine profiles, with the reduction of those with an anti-inflammatory and anti-cancerous activity, lead to an increase in mitogenic signals, a decrease in cell apoptosis and an increase in pro-angiogenic signals, which are risk factors for the development of CRC [67,130]. The role of the components of the ghrelin system produced by visceral AT is primarily suggested in association with the regulation of GH/IGF-1 axis and its downstream signalling pathways [130].

Unfortunately, no meta-analysis on the role of ghrelin as an obesity-related adipokine in CRC has been performed so far, mostly due to the small number of reports on the subject [131].

## 4. Genetic/Epigenetic Alterations of the Ghrelin System in CRC

Some interesting trials examined the association between common genetic variants in the genes encoding ghrelin (*GHRL*) and its receptor (*GHSR)* and colorectal cancer risk [132,133]. The first case-control study regarding single nucleotide polymorphisms (SNPs) in the *GHRL*, and the *GHSR*, and CRC risk, was published in 2010. In two unrelated populations (Czech Republic, Germany), two SNPs, namely SNPs rs27647 and rs35683, were found to be associated with a lower CRC risk [132]. In contrast, in an Iranian population study on ghrelin rs26802 genotyping, no significant difference was observed in terms of genotype or allele frequencies between patients with CRC and controls [133]. Moreover, two meta-analyses showed no statistically significant association between CRC and the polymorphisms of the studied ghrelin system components [134,135].

As for epigenetic changes in the ghrelin system, there is one report so far that has showed significant hypermethylation of *GHSR* in CRC tissues compared to normal mucosa, which was not accompanied by significant changes in *GHRL* methylation. *GHSR* hypermethylation was detectable as early as the adenoma stage, and persisted in later stages regardless of clinical factors (e.g., age, sex, anatomical location, grading, MutL homolog 1 (MLH1) deficiency, etc.) [136].

It is important to note that *GHRL* is not a classical oncogene, such as genes crucial in the development and metastasis of CRC (e.g., *APC*, *PIK3CA*, *KRAS*, *TP53*, *SMAD4*, and *BRAF*) [65].

The oncogenic role of the ghrelin system in activating processes associated with CRC carcinogenesis (e.g., cell proliferation, migration, invasion, and apoptosis), and the signalling pathways that are responsible for tumour growth and progression, will be discussed later in this paper.

## 5. The Ghrelin System in Clinical Studies—A Continuous Lack of Evidence of a Significant Role in Development and Progression of Colorectal Cancer

### 5.1. Serum/Plasma Concentrations of Ghrelin in CRC

Diagnostic and prognostic assessment of serum ghrelin concentrations in CRC patients has already been conducted by more than a dozen groups of investigators in different populations, with the number of such studies steadily increasing. Part of the work was carried out using a radioimmunoassay (RIA), while others were based on the enzyme linked immunosorbent assay (ELISA) method. Their findings are controversial, as also highlighted by other reviews [6,56,74].

Some investigators have shown reduced ghrelin levels in CRC patients compared to control groups [137,138,139,140], or mild lesions [141], and colon cancer (CC) patients in relation to rectal cancer (RC) [139]. These studies showed different correlations between ghrelin levels and clinical data, tumour histology or location. Thus, differences were observed between ghrelin levels and tumour location (lower in left colon), *H. pylori* infection (lower), or tumour stage (decreasing with increasing stage) [137]. Similarly, Murphy et al., in the first prospective report on the subject, observed an association between low ghrelin levels assessed in blood samples taken 10 years prior to tumour development and an increased risk of developing CRC [142]. Other papers that observed reduced ghrelin levels in CRC compared to controls either showed no significant correlation with clinical data [138], an inverse correlation between ghrelin levels and severity of epigastric bloating in CC [139], or weak negative correlations between BMI and homeostatic model assessment–insulin resistance (HOMA-IR) in patients with RC [140].

Two studies have shown a definite increase in serum levels of this peptide in patients with CRC vs. control group [143,144]. There were also positive correlations of this concentration increase with tumour staging and grading [143], as well as with tumour location in CC, and age in patients with RC [144]. These studies suggested local production of ghrelin by colon tumours [143].

In CRC patients with cachexia, either significantly higher mean ghrelin levels were observed compared to the group without such a condition [59,145], or no quantitative differences were recorded between both groups [146]. The discussion considers, among other things, the individual BMI range of the patients studied. Waseem et al. found that ghrelin levels correlate with the metabolic state of the patient rather than being a predictor of advancing tumour stage [59].

In a recent study describing various GI tract tumours (including CRC), positive correlations were found between levels of active ghrelin, IL-6 and energy metabolism, and negative with food intake rate, which according to authors could suggest ghrelin resistance. This study confirmed an increase in inflammatory cytokines with the progression of GI tract cancers, suggesting their possible link with decreased fat-free mass (FFM) and increased energy metabolism. However, increased levels of active ghrelin failed to compensate for cachexia in the studied patients [147].

As mentioned, a prospective study on a large group of patients (over 500 patients, Finnish smokers) on the role of serum ghrelin concentrations as a risk factor for the development of CRC was initiated by Murphy et al. [142] Low levels of this peptide 10 years before CRC diagnosis were significantly correlated with an increased risk of developing CC and RC. Interestingly, in people with longer cancer development times (more than 20 years after the blood sample was taken), low levels of this hormone taken so early were instead correlated with a significant reduction in the risk of developing cancer [142]. However, a discussion was presented by Sundkvist et al., who achieved these results through their own observations on a group of 60 patients with CRC from a similar human population (Scandinavian population) which did not confirm an association between reduced ghrelin levels and increased CRC risk in the years before diagnosis. They observed unchanged ghrelin levels in CRC patients compared to controls, both in samples taken less than 5 years and more than 10 years after tumour development [148].

To summarise, generalising the results and drawing conclusions in such research is made difficult by several factors. Differing results may occur due to (1) differences in test methodology (different types of ghrelin tested, ELISA vs. RIA); (2) heterogeneous patient groups, including often small numbers of serum/plasma samples from CC and RC or lack of control samples; and (3) coexistence of unspecified hormonal factors affecting ghrelin production in this part of the GI tract at different stages of colon carcinogenesis.

Most of the tests were performed using ELISA, which increases specificity and results in less cross-reactivity in ghrelin concentrations testing compared to RIA [148]. One group of researchers evaluated patients after surgical excision of cancer [139], which is difficult to compare with groups before such treatment (most studies). However, there are also works in which ghrelin levels were assessed before and after CRC treatment, showing, similarly to Zygulska et al., a decrease in hormone levels after tumour resection [144]. Evaluation of ghrelin concentrations prior to surgical treatment of CRC, studied by this group, resulted in additional clinical correlations not supported by other work [144]. Only one group investigated the relationship between ghrelin levels and CRC patient survival, showing no significant relationship [143].

In conclusion, studies of serum ghrelin concentrations in CRC demonstrate that this hormone does not meet the conditions for a good serum biomarker of the risk of CRC development and/or prognosis [142,148]. Regarding the demonstrated reduced ghrelin levels in CRC, it is suggested that low ghrelin concentrations play a role in creating a metabolic proinflammatory environment in the early stages of CRC development, resulting in enhanced tumour growth. In contrast, it is also possible to envision a scenario in which the reduced serum levels of ghrelin in CRC patients are a secondary occurrence, resulting from the inhibitory effect of other tumour progression-associated factors/hormones on its production [142].

A comparison of circulating ghrelin levels in different populations with CRC is presented in Table 1.

### 5.2. Tissue Expression of the Ghrelin System in CRC

Similarly to normal colonic epithelial cells [93], colorectal adenoma [96] and colorectal adenocarcinoma cells express ghrelin and its receptors (GHS-R1a, and GHS-R1b) [59,61,95]. Colorectal adenoma and CRC tissues are characterised by a higher local expression of ghrelin system components compared to normal colon mucosa. All the above-mentioned components of the ghrelin system show a predominantly cytoplasmic, rather than nuclear, pattern of IHC expression [59,61,95,96]. A recently published (2021) pioneering work on tissue expression of ghrelin and ghrelin receptors (without specific receptor typing) in colorectal adenoma illustrates that a strong response to ghrelin was 7-fold more frequent in high-grade adenomas vs. adenomas with low-grade dysplasia. Furthermore, in an adenoma of high-grade dysplasia, the most significant positive correlation between ghrelin and its receptor expression was observed. According to the authors, these results indicate an important role for ghrelin in the progression of colorectal dysplasia, although further studies are required to understand the mechanisms of cell proliferation and malignant transformation [96].

In turn, results regarding the correlation between tissue expression of the ghrelin system with clinical data in colorectal adenocarcinoma are divergent. Some show higher expression of ghrelin and GHS-R1b, and reduced GHS-R1a in more severe stages of CRC. Similar correlations were observed between the expression of both receptors and grading. However, when it comes to ghrelin expression and grading, higher expression vs. control samples was observed only in well- and moderately differentiated tumours. Interestingly, a complete loss of IHC signal for ghrelin and its receptors was observed in poorly differentiated CRC (highly malignant tumours) [59]. Similarly, Wang et al. observed negative correlations between GHS-R1a expression and grading [95]. In turn, no significant correlations between ghrelin/GHS-R1a expression and grading could be demonstrated in another publication [61]. Only Waseem et al. obtained a high positive correlation between tissue ghrelin expression and BMI of CRC patients [59].

The discrepancies in the results obtained by different authors in tissue material from patients with adenoma and CRC may occur for various reasons, including the number and size of CRC samples tested, the primary antibodies used (rabbit/goat anti-human), all being polyclonal and not monoclonal, the suppliers they were sourced from (e.g., Chemicon, Phoenix Pharmaceuticals, Santa Cruz, ABCAM), the lack of differentiation between the types of ghrelin receptors tested, different scoring systems used to assess the intensity of expression, and more.

A summary of the results on tissue expression of ghrelin system components in CRC and colorectal adenoma, with their possible role in pathogenesis, diagnosis, and prognosis, is presented in Table 2.

At the tissue level, using RNA extraction and quantitative RT-PCR (qRT-PCR) significantly reduced GHRLOS expression, which was detected in the tissues of nearly 55% of CRC patients compared with adjacent non-cancerous colon tissue. It was reported that a decreased expression of GHRLOS is an independent prognostic marker of poor outcomes, namely disease-free survival (DFS) and overall survival (OS) (HR = 2.0., 95 CI = 1.42–3.88, and 1.96, 95% CI = 1.34–2.86). GHRLOS may act as a tumour suppressor during CRC development, and downregulation of its expression may facilitate tumour progression and metastasis. However, the exact mechanism of GHRLOS action, and the influence of other transcription factors directly regulating its expression and downregulation in CRC, are still an open question [149].

In conclusion, the study of tissue expression of peptides from the ghrelin system using only the IHC technique is of limited use. Nonetheless, validation of these findings using other models and research techniques (cultured cells, colorectal tumour xenograft mouse model, qRT-PCR) confirms the local and, often, increased production of ghrelin and its receptors in the cells of this tumour, as will be described later in this paper. A prognostic significance in CRC was demonstrated only for lncRNA (GHRLOS) expression.

**Figure 2:**
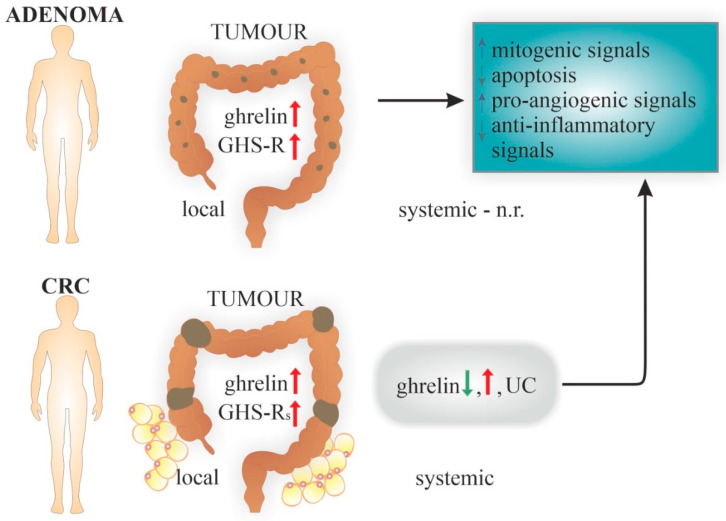
Potential role of both local expression and systemic levels of the ghrelin system in the pathogenesis of colorectal cancer (CRC). [↓/↑-reduced/increased expression/level; AG-acylated ghrelin; GHS-R(s)-ghrelin receptor(s); UC-unchanged; n.r.-not reported].

A schematic of the potential role of the ghrelin system in colorectal cancer (CRC) pathogenesis, based on in vivo studies, is presented in Figure 2.

## 6. Studies on Potential Mechanisms of Ghrelin System Components in Colorectal Carcinogenesis

### 6.1. In Vitro Studies

Tumour cell lines derived from colorectal tumours with different degrees of differentiation are characterised by the production (mRNA, protein) of endogenous (native) ghrelin and both its receptors. This expression is higher than in normal intestinal epithelial cells [59,60]. Caco2 colorectal adenocarcinoma cells also showed high levels of GHRLOS expression [125]. GHRLOS completely overlaps the ghrelin gene, and hence may also have a major effect in regulating the ghrelin axis [8].

The pro-proliferative properties of both native ghrelin [59,60], as well as exogenous ghrelin injected into cultures of normal epithelial cells and transformed colonocytes, were already described [61]. An increase in invasiveness, cell migration [59,60], and cell viability was also observed [61,150,151]. Administration of exogenous ghrelin also resulted in increased endogenous ghrelin mRNA production and weaker expression of both of its receptors in HCT116 cells, with a gradual decrease in production after prolonged peptide administration (18 and 24 h). This could indicate the presence of negative feedback mechanism in these cells, triggered by exogenous ghrelin [152].

The effect of ghrelin on tumour cell growth in various GI tract cancers was discussed by a number of authors [6,55,72]. The majority of publications describe proliferogenic effects of ghrelin system components in this tumour type [153,154,155]. In CRC cells, enhancement of proliferation and cell cycle promotion could be mediated via adenylate cyclase (AC)-independent epidermal growth factor receptor (EGFR) trans-activation and PI3K/Akt phosphorylation [60]. Activation of Ras/PI3K/Akt/mTOR signalling was also demonstrated in the study of Lien et al. [62]. This mechanism was confirmed in the study based on the *GHSR1a* knockdown model, in which a decrease of Ras/PI3K/Akt pathway activity was demonstrated, correlating with an increase in the level of phosphatase and tensin homolog deleted on chromosome ten (PTEN) protein. The authors summarise that the regulation of the PTEN/PI3K/Akt pathway is associated with GHS-R1a-induced proliferation in poor-differentiated SW480 cells [61].

The only work showing a rather weak antiproliferative or antineoplastic effect of ghrelin was conducted in MC38 murine colon cancer cells. Application of a GHS-R1a antagonist (D-Lys-GHRP-6) resulted in biphasic activity, with strong inhibition and weak stimulation of cell growth in vitro. A stronger inhibitory effect on MC38 cell growth was obtained when D-Lys-GHRP-6 was administered together with fluorouracil (FU) and UnAG. In turn, UnAG alone had a rather weak growth inhibitory effect (8–10%) as compared to the controls [156].

Differential involvement of the ghrelin system in CRC cell apoptosis was also described [150,152]. Downregulation of 5-FU-induced apoptosis in HT-29 cells through regulation of the Bcl-2/Bax system was described in one study [150], while an induction of HCT116 apoptosis following exogenous ghrelin administration via a mechanism of ubiquitin-proteasome system inhibition and increased autophagy was reported in another [152].

In conclusion, in vitro model studies using different CRC cell lines strongly confirm the local production of ghrelin and its receptors, and the mainly pro-proliferative properties of the whole system. Ghrelin also increased the invasion and migration of cancer cells, which could potentially play a role in cancer progression.

### 6.2. Animal In Vivo Models

While the potential role of the ghrelin system has also been studied in mouse models of colorectal carcinogenesis, such works are relatively sparse [61,157]. Significant reductions in tumour weight were demonstrated in *GHSR1α* knockdown SW480 mouse xenograft tumours compared to tumours from negative controls in a study of Liu et al. [61] Moreover, interesting research on the administration of exogenous AG or in the absence of endogenous ghrelin (*Ghrl* deletion) was performed in two mouse models of colon carcinogenesis—genetic (*Apc*^Min/+^ mice) and inflammation-associated (azoxymethane (AOM)/DSS). In inflammation-induced colitis, administration of exogenous ghrelin significantly inhibited colon tumour formation. In contrast, ghrelin administration had no effect on the number of intestinal tumours forming in *Apc*^Min/+^ mice. While the absence of endogenous ghrelin did not affect the incidence of intestinal tumours in both AOM/DSS-treated and *Apc* mutant mice, the size of tumours was larger in the *ghrl^(−/−)^* colon than in the AOM/DSS model. Interestingly, no tumour-promoting effect was observed after ghrelin administration in any of the models [157].

A chronological summary of the major findings in in vitro and mouse models of the study regarding the involvement of the ghrelin system in the basic mechanisms of colorectal carcinogenesis is presented in Table 3.

## 7. Therapy Using Ghrelin System Components in CRC-Associated Cachexia and Sarcopenia

Patients with cancer (including CRC) are at greater risk of losing muscle mass through two different mechanisms: sarcopenia, defined as an age-related decrease in muscle mass via changes in muscle synthesis signalling pathways, and/or cachexia, defined as cytokine-mediated muscle and fat tissue degradation [158,159]. There is a growing understanding of the causative factors of sarcopenia, including metabolic dysregulation, intestinal dysbiosis, diet, and lifestyle in ageing people [160]. Criteria are being defined for impaired food intake and CRP values, which may improve the diagnosis and classification of cancer-related cachexia [161]. An in vitro model has been developed that can be used to study tumour-induced myoblast apoptosis. These results suggest the possibility of using both forms of ghrelin (AG and UnAG) in the treatment of cancer cachexia [159]. As there is as yet no universal therapy for both of these multifactorial syndromes, attempts are also being made to use anabolic-orexigenic agents based on ghrelin system components.

Therapeutic attempts of ghrelin and ghrelin receptor agonists used in cancer cachexia [152,162,163,164] and chemoprevention of inflammation-associated CRC carcinogenesis in animal models [157], have given rise to the idea of including such a therapy in humans. The effectiveness of cancer cachexia treatment is determined by a number of basic (primary) parameters, e.g., food intake, weight gain, lean body mass (LBM) gain, fat mass gain, and survival in the setting of cancer cachexia (reviewed in: [165]). Additional (secondary) end points assessed in the treatment of cachexia also comprise changes in quality of life, general nutritional status of patients or serum biomarkers of nutritional status (e.g., IGF-1, IGFBP-3, and prealbumin) [166].

Studies and/or clinical trials report the beneficial effects of ghrelin [167,168] and an agonist for the ghrelin receptor (anamorelin) in the treatment of cancer-associated cachexia [166,169,170,171] and cancer-associated sarcopenia (reviewed in: [172]). In such trials, subcutaneously administered synthetic ghrelin [167], or natural ghrelin, was used [168]. Although these studies are based on a small number of patients with cachexia, good tolerability and safety of ghrelin administered in such way has been demonstrated in patients with advanced disease (most with metastatic cancer) and with cancer cachexia in pancreatic, head and neck, lung, and gastrointestinal cancer. Moreover, positive effects of ghrelin on food intake, stable muscle mass or muscle growth and high exercise tolerance have been observed [168]. Subcutaneously administered ghrelin resulted in an increased appetite, improved energy balance, attenuated catabolism, and supported host metabolism [167].

An oral ghrelin-receptor agonist with appetite-enhancing and anabolic properties, known as anamorelin hydrochloride, was studied in both healthy volunteers and cancer patients with cachexia [169,173]. In healthy volunteers, increases of GH, IGF-1, IGFBP-3, and body weight were observed, with good tolerability and selectivity [173]. Treatment of patients with anorexia-cachexia-cancer syndrome for 12 weeks resulted in a favourable clinical response profile, although some adverse effects were also observed [169]. The orally administered anamorelin (ANAM tablets) was also studied in Japanese patients with CRC, gastric, and pancreatic cancer. It has been shown to have beneficial effects on advanced and unresectable gastrointestinal cancer (including CRC) [166]. Its use was well tolerated and improved anorexia and patient nutritional status, resulting in increased LBM and body weight in patients with cancer cachexia. Importantly, other non-clinical and clinical studies indicate that ANAM promotes secretion of GH, IGF-1, and IGFBP-3 but not tumour growth [169,173].

Anamorelin is a drug approved (December 2020) only in Japan (not Europe) for the treatment of cancer cachexia in multiple solid tumours, including CRC-associated cachexia [166,170]. Recent studies have confirmed the effect of anamorelin on maintaining and increasing LBM and body weight, as well as improving anorexia. The efficacy and safety of anamorelin in treating cancer-related cachexia was confirmed [171].

Therapeutic options based on the ghrelin system in CRC-associated cachexia are summarised in Table 4.

Sarcopenia occurs in 12–60% of CRC patients and appears to be a risk factor for multiple complications after CRC surgery [174]. It could also negatively affect OS, DFS, recurrence-free survival (RFS), and cancer-specific survival (CSS) in these patients. In addition, patients with sarcopenia appear to be susceptible to the toxic effects of chemotherapy. Therefore, the use of ghrelin may help preserve muscle mass in metastatic CRC (reviewed in: [172]). The effects of ghrelin and its analogues (anamorelin) stimulate appetite and muscle anabolism, indicating the potential importance of the ghrelin system in alleviating CRC-associated sarcopenia. The understanding of GHS-R signalling and the development of new drugs and non-peptide agonists of this receptor (e.g., ibutamorene) could also be an important factor in the treatment of sarcopenia in cancer. Recent studies reveal the molecular basis of the binding of ghrelin and ibutamorene to GHS-R [175].

## 8. Concluding Remarks and Future Perspectives

In the normal large intestine, different ghrelin system components are detected in small amounts and are mainly implicated in colon motility. In obesity, a risk factor for CRC, ghrelin secretion in visceral adipocytes is increased. This excess may result in increased mitogenic signalling, decreased cell apoptosis and increased pro-angiogenic signalling. One pathway that is important in these mechanisms is the GH/IGF-1 axis and its downstream signalling pathways. However, this matter requires continued large-scale prospective studies to better understand the role of the ghrelin system in this pathology.

Genetic alterations of *GHRL/GHSR* in CRC usually occur in the form of SNPs and are not significant risk factors for CRC development or progression. However, further investigation of the contribution of the ghrelin system genetic alterations in patients with many additional risk factors for CRC development (including obesity, MetS, and T2D) is required. The findings on epigenetic alterations of the ghrelin system (hypermethylation of *GHSR* in adenoma) are encouraging, especially in terms of their clinical utility in CRC.

Although there is an increasing number of studies on serum ghrelin concentrations, and tissue expression of components of the ghrelin system (ghrelin, GHS-Rs, GOAT) in CRC patients, still none of them meet the conditions for a good biomarker of development risk and/or prognosis of this tumour. Prognostic significance in CRC was demonstrated only for the expression of lncRNA (GHRLOS), which functions as a tumour suppressor during the development of this cancer. The role of the perinuclear localization of UnAG in the context of colon carcinogenesis is interesting, although it is not yet fully understood.

In vitro models on CRC cells confirm the local production of ghrelin/GHS-Rs and the mainly pro-proliferative properties of this system. This hormone also increased the invasion and migration of cancer cells. However, administration of exogenous ghrelin in various CRC models (including inflammation-associated mouse model) had no direct colon carcinogenesis-promoting effect. The potential significance of the effects observed in vitro on CRC progression in vivo remains to be elucidated.

Further research is needed to link the ghrelin system to IBD mechanisms (especially ulcerative colitis (UC)), as important risk factors for CRC. Investigating the molecular mechanisms of UC-associated CRC, regarding the anti-inflammatory effects of the ghrelin system in multiple tissues may allow for the development of better therapeutic approaches.

The use of ghrelin and an agonist for the ghrelin receptor (anamorelin) in the treatment of cancer-associated cachexia and sarcopenia has been attempted with good results. Anamorelin is expected to provide a new therapeutic option for cancer cachexia, for which no effective treatment has been available to date.

## Figures and Tables

**Table 1:** Circulating Concentrations of Ghrelin in CRC Patients.

Characteristics of the Patient	Material and Method	Level of Ghrelin	Correlations with Clinical Parameters	Refs.
n = 40 BC and CRC, including n = 12 CRC cachectic and n = 14 noncachectic patients; no control group; Israeli population	fasting blood samples; RIA (pg/mL)	↑in all cachectic vs. noncachectic patients	(i) in all patients BMI loss was a significant independent predictor of ghrelin levels; (ii) stronger correlation with cachexia in woman vs. man	[145]
n = 78 GC and CRC, including n = 20 CRC (n = 7 CRC cachectic and n = 13 noncachectic patients); n = 24 C; Chinese population	fasting blood samples; RIA (pg/mL)	CRC vs. C*^NS^*; cachectic CRC vs. noncachectic patients*^NS^*	no correlation between plasma ghrelin and other hormones, CRP, body composition parameters, and tumour stage	[146]
n = 29 CRC; n = 50 C, Italian population	fasting serum samples; RIA (pg/mL)	↓vs. C	(i) lower levels in left colon tumours and with *H. pylori* infection; (ii) ↓from earlier to later tumour stages	[137]
n = 110 CRC; Pakistan population	fasting blood samples; total plasma ghrelin; RIA (pg/mL)	↑in cachectic patients vs. C	no correlation with age, BMI, grade/stage of CRC	[59]
n = 126 CC; n = 36 C; Turkey population	fasting serum samples; RIA (pmol/L);	↓vs. C	no correlation with clinical parameters	[138]
n = 20 CRC before and after therapy; n = 20 benign group before and after therapy; Turkey population	serum levels; ELISA (ng/mL)	↓in CRC vs. benign group (both before therapy); in CRC before therapy vs. after therapy*^NS^*	nt	[141]
n = 95 CRC; n = 39 C; Greek population	fasting plasma samples; total plasma samples; ELISA (pmol/L)	↑vs. C	↑, ♣, ♦; no correlation between total plasma levels and survival	[143]
n = 30 CC; n = 30 RC after surgical treatment; no cachectic patients; n = 30 C; Poland’s population	fasting plasma samples; ELISA (pg/mL)	↓in CC vs. RC and vs. C	(−)correlation with severity of epigastric bloating in CC	[139]
n = 284 CC; n = 239 RC; n = 523 C; Scandinavian population (Finnish smokers)	nested case-control study; total serum samples; prospective study; RIA (pg/mL);	low level associated with ↑CRC risk occurring within 10 yrs of blood draw; ↓CRC risk in cancers occurring >20 yrs after blood draw	smoking (either intensity or duration) did not alter the observed associations	[142]
n = 33 CC; n = 27 RC; n = 60 C; Scandinavian population	fasting plasma samples within 5 yrs preceding diagnosis of the cases; prospective study; ELISA (pg/mL)	CRC vs. C (<5 yrs)*^NS^* CRC vs. C (>10 yrs)*^NS^*	plasma levels not associated with CRC risk	[148]
n = 24 CC; n = 26 RC; n = 69 C; Chinese population	serum samples, ELISA (pg/mL)	↑preoperative levels vs. C; the highest levels in RC; ↓after tumour resection	(i) perioperative levels: (+)correlation with tumour location in the CC, and age with RC (higher in >60 yrs vs. <60 yrs); (ii) postoperative levels: ↑ in CC in the descending vs. ascending colon; ↑in NRS2002 score ≥ 3 vs. score < 3 in RC	[144]
n = 19 CRC and other GI tract cancers (oesophageal, GC); Japan population	AG and UnAG, (fmol/mL)	↑level of AG in stage IV compared with stage III in all GI tract cancers	(i) (+)correlation with IL-6 level and energy metabolism; (ii) (−)correlation with food intake rate	[147]
n = 82 CRC; n = 88 C; Iranian population	fasting plasma samples; ELISA (pg/mL)	↓vs. C	week (−)correlation with BMI and HOMA-IR in RC	[140]

[↓/↑—decrease/increase level; (+)/(−)—positive/negative; ♣—significant association between ghrelin and degree of cancer differentiation; ♦—association between ghrelin and more advanced clinical or TNM stage of cancer; AG—acylated/active ghrelin; BC—breast cancer; C—control; BMI—body mass index; CC—colon cancer; CRC—colorectal cancer; CRP—C-reactive protein; GC—gastric cancer; ELISA—the enzyme linked immunosorbent assay; HOMA-IR—homeostatic model assessment-insulin resistance; *H. pylori*—*Helicobacter pylori*; NRS2002—Nutritional Risk Screening 2002; NS—statistically nonsignificant; nt—not tested; RC—rectal cancer; refs.—references; RIA—radioimmunologic assay; TNM—tumour, node, metastasis; UnAG—des/unacylated ghrelin; yrs—years].

**Table 2:** Tissue Expression of Ghrelin System Components in CRC and Colorectal Adenoma.

Material and Methods	Ghrelin System			The Main Results of the Study	Refs.
	Ghrelin	GHS-R1a	GHS-R1b		
n = 12 CRC and C; TMA; IHC	nt	↑vs. C; ↑Cyt vs. N	nt	(i) negative correlation with ♣,♦; (ii) ↑expression in patients with lower weight loss vs. higher weight loss	[95]
n = 110 CRC and C; IHC	↑vs. C; N, Cyt	↑vs. C; Cyt	↑vs. C; Cyt	(i) ↑vs. C in advanced stage; (ii) gradually ↑GHS-R1b expression with advancing tumour stage; (iii) (−)correlation of GHS-R1a with ♦; (iv) ↑vs. C in well- and moderately-differentiated CRC; (v) ↑GHS-R1b and ↓GHS-R-1a in low-grade tumours; (vi) loss of ghrelin and GHS-Rs in highly undifferentiated CRC; (vii) (+)correlation between ghrelin and BMI	[59]
n = 150 CRC and C; IHC	↑vs. C; Cyt	↑vs. C; Cyt	nt	no correlation between ghrelin and/or GHS-R1a expression and tumour grades	[61]
n = 92 colorectal adenoma; adjacent colon tissue (C); IHC	↑vs. C; Cyt	↑GHS-R in adenoma vs. C		(i) 7× more common ↑ghrelin in high-grade vs. low-grade adenoma; (ii) the most significant correlation between ghrelin and GHS-R in adenomas with high-grade dysplasia	[96]

[↓/↑—decrease/increase level; (+)/(−)—positive/negative; ♣—significant association between ghrelin and degree of cancer differentiation; ♦—significant association between ghrelin and more advanced clinical or TNM stage of cancer; BMI—body mass index; C—control, normal epithelial cells; Cyt—cytoplasmic localization; CRC—colorectal cancer; GHS-R1a/R1b—ghrelin receptor 1a/1b; IHC—immunohistochemistry; N—nuclear localization; nt—not tested; refs.—references; TNM—tumour, node, metastases; TMA—tissue microarray].

**Table 3:** The Potential Role of the Ghrelin System in Colorectal Carcinogenesis—in vitro and mouse model studies (*) on exogenous ghrelin in cancer.

Model of the Study	The Components of Ghrelin System						The Main Mechanisms of Action	Refs.
	Ghrelin		GHS-R1a		GHS-R1b			
	mRNA	Protein	mRNA	Protein	mRNA	Protein		
well differentiated CCs (SW-48)	(+++)	(+++)	(++)	(++)	(+++)	(+++)	↑↑cell proliferation/invasion/migration	[59]
poorly differentiated CCs (RKO)	(+++)	(+++)	(++)	(++)	(+++)	(+++)	↑↑cell proliferation/invasion/migration	
normal human colonocytes	(+)	(+)	(+)	(+)	(+)	(+)	do not proliferate	
hCCs (HT-29); eG	nt	nt	nt	nt	nt	nt	(i) ↑cell viability; (ii) ↓5-FU-induced apoptosis via regulation of Bcl-2/Bax ratio	[150]
murine colon cancer MC38 cells; eG (hAG, hUnAG)	nt	nt	nt	nt	nt	nt	(i) hG-dose-response for anti-proliferative action with the synergistic effect of hUAG and GHS-RA; (ii) hUnAG-↓ or ↑antineoplastic effect of GHS-RA; (iii) biphasic activity of GHS-RA (↓↓/↑of cell growth)	[156]
hCCs (HCT116); eG	(+),↓at 18 and 24 h treatment	nt	(+),↓at 18 and 24 h treatment	nt	(+),↓at 18 and 24 h treatment	nt	(i) negative feedback triggered by eG; (ii) direct ↓of 20S proteasomes; (iii) ↑of apoptosis by ↓ubiquitin-proteasome system and by ↑autophagy	[152]
normal human intestinal cells (FHs74Int); eG (AG, UnAG)	(+)	(++)	(++)	(++)	(++)	(++)	(i) ↑cell proliferation in all cells under both isoforms of eG, ↓cell proliferation in higher doses of eG; (ii) ↑of cell cycle progression via PI3K/Akt pathway and EGFR trans-activation both converging to ERK 1/2 phosphorylation	[60]
RKO, hCRCs (Caco-2); eG (AG, UnAG)	(+)	(++); Cyt, N	(++)	(++); M	(++)	(++); M		
HCT116 cells; eG	nt	nt	nt	nt	nt	nt	(i) ↑in cell viability vs. untreated cells; (ii) ↑↑in cell viability of cells treated solely with eG vs. the groups treated with the eG + melatonin, and leptin + melatonin	[151]
AOM/DDS-induced inflammation-associated colon carcinogenesis and *Apc*^Min/+^ mouse model; eG	(++)	nt	(++)	nt	(++)	nt	(i) ↓↓in tumour incidence in AOM/DDS colitis but not in *Apc*^Min/+^ model; (ii) no tumour-promoting effect in either model; (iii) the chemopreventive effect of inflammation-associated colorectal carcinogenesis; (iv) loss of ghrelin did not affect the incidence of intestinal tumour formation in either model	[157] *
normal colon epithelial cells (NCM460)	nt	(+)	nt	(+)	nt	nt	(i) in KD model-↓cell viability vs. blank/scrambled C regardless of the eG application; (ii) in KD model-↑PTEN, ↓PI3K/AKT pathway and promoting the release of p53 in SW40 cells	[61] *
Caco-2 cells, SW480 cells; eG	↑vs. NCM460	↑vs. NCM460	SW480 > Caco-2	↑vs. NCM460	nt	nt		
colorectal tumour xenograft mice with GHS-R1a KD	nt	nt	nt	nt	nt	nt	(i) ↓tumour weight vs. blank/negative C tumours; (ii) ↓Ki-67(+) cells vs. blank/scrambled C; (iii) ↑PTEN-positive cells vs. other groups	
HT-29 cells; eG	nt	nt	nt	nt	nt	nt	(i) ↑cell proliferation via Ras/PI3K/Akt/mTOR signalling; (ii) time-dependent ↑Ras activity	[62]
hCCs (HCT-15); eG	nt	nt	nt	nt	nt	nt	↑cell proliferation	

[(↑↑)↑—(significant/strong) increase/promotion/induction/; (↓↓)/↓—(significant/strong) decrease/inhibition; (+)—minimal expression; (++)—expression; (+++)—overexpression; AKT/Akt—serine/threonine-protein kinase or protein kinase B (PKB); AOM—azoxymethane; APC/^Min+^—adenomatous polyposis coli/multiple intestinal neoplasia^+^; C—control; Cyt—cytoplasm; DDS—dextran sodium sulphate; eG—exogenous/synthetic ghrelin; EGFR—epidermal growth factor receptor; ERK1/2—extracellular signal-regulated kinase 1/2; 5-FU—5-fluorouracil; GHS-RA—ghrelin receptor type 1a antagonist; hAG—human acylated ghrelin; hCCs—human colon cancer cells; hCRCs—human colorectal cancer cells; hUAG—human unacylated ghrelin; KD—knockdown; M—membranous localization; mTOR—the mammalian target of rapamycin, protein kinase; N—nuclear localization; nt—non tested; PI3K-PKC—protein kinase C; PTEN—phosphatase and tensin homolog deleted on chromosome ten; Ras—“Rat sarcoma virus” protein; refs.—references].

**Table 4:** Therapeutic Options Based on the Ghrelin System in Colorectal cancer (CRC)-associated Cachexia.

Name of Targeted Agents	Agent Characteristics and Doses	Group/Model of the Study	Effects	Stage of Development	Refs.
Synthetic ghrelin	~13 µg/kg or 0.7 µg/kg daily s.c. for 8 wk	solid GI tract tumours; unselected weight-losing cancer patients	(i) supports host metabolism; (ii) improves appetite; (iii) attenuates catabolism	randomised, double-blind study (National Clinical Trial no. NCT00681486)	[167]
Anamorelin hydrochloride (RC-1291 HCl, tabl. 50 mg)	synthetic peptide agonist of GHS-R; 50 mg or placebo once-daily for 12 wk	44 patients with CC and n = 38 placebo group	(i) ↑LBM; (ii) a favourable clinical response profile in patients with cachexia	phase 2, multicentre, placebo-controlled, double-blind trials; ClinicalTrials.gov, numbers NCT00219817 and NCT00267358	[169]
Anamorelin (ONO-7643; ANAM, tabl. 100 mg)	agonist of GHS-R; once daily over 12 wk	50 Japanese patients with advanced and unresectable CRC, GC, and PC	rapid ↑LBM and BW in patients with advanced GI tract cancer who had CC	multicenter, open-label, single-arm study	[166]
Anamorelin hydrochloride (ADLUMIZ, tabl. 50 mg)	selective agonist of GHS-R1a	humans	(i) maintains and ↑LBM and BW; (ii) improves of anorexia; (iii) the efficacy against CC	phase III study for CRC, GC, and PC	[171]
Promising trials on animal models or in vitro systems					
GHRP-2	agonist of GHS-R; s.c. 10 μg/mouse daily; 5-FU+GHRP-2; 5-FU alone	BALB/c female colon tumour-bearing mice (CT26 colorectal adenocarcinoma cells)	5-FU+GHRP-2 improved appetite in tumour-bearing mice with anorexia/cachexia syndrome in early stage	may improve the efficacy of therapy and the quality of life thank to the amelioration of their nutritional state	[162]
Exogenous ghrelin	1–10 µM of mostly UnAG	human colon cancer HCT116 cells	(i) direct ↓of 20S proteasomes; (ii) ↑of apoptosis by ↓ubiquitin-proteasome system and by ↑autophagy	the proteasome as target for cancer therapy	[152]
Exogenous ghrelin	i.p. injection of AG (3 nmol/day)	AOM/DDS and *Apc*^Min/+^ mouse model of CRC	(i) the chemopreventive effect of inflammation-associated CRC; (iv) loss of ghrelin did not affect the incidence of tumour formation	in vivo experimental evidence for the usefulness of ghrelin in the chemoprevention of inflammation-associated CRC carcinogenesis	[157]
HM01	agonist of GHS-R; 10 mg/kg and 2 × 20 mg/kg/day orally until day 20	mice bearing CT26 cells; healthy mice	(i) ↑BW, fat mass, neuronal hypothalamic activity in healthy mice; (ii) ↑food intake, BW, fat mass, SM mass, bone mineral density (iii) ↓energy expenditure in tumour-bearing mice; (iv) capable to counteract CC without interfering with cytokine or E3 ligase signalling	counteracts cachectic BW loss under inflammatory conditions and is a promising compound for the treatment of CC in the absence of severe anorexia	[163]
Exogenous ghrelin	AG, UnAG-0.8 mg/kg i.p. twice daily from day 14, when the mice presented signs of cachexia	mice bearing CT26 cells	(i) both ghrelins-↓calpain activity in SM of cachectic mice; (ii) improved tumour-free BW, grip strength, muscle mass, and nutritional state; (iii) ↓serum TNF-α, ↑Akt activity, and ↓atrogin-1 in SM	contributed to the development of an AG/UnAG-based therapy for CC	[164]

[↑,↓—increase (up-regulation)/decrease (inhibition)/expression/level; AG—acylated ghrelin; AOM—azoxymethane; APC/^Min+^—adenomatous polyposis coli/multiple intestinal neoplasia^+^; BW—body weight; CC—cancer cachexia; DDS—dextran sodium sulphate; GC—gastric cancer; GHS-R—ghrelin receptor; GHRP-2—ghrelin agonist growth hormone releasing peptide; GI—gastrointestinal; i.p.—intraperitoneally; LBM—lean body mass; PC—pancreatic cancer; s.c.—subcutaneously; SM—skeletal muscle; tabl.—tablets; TNF-α—Tumour Necrosis Factor α; UnAG—unacylated ghrelin; wk—weeks].

## Data Availability

Not applicable.

## Abbreviations

AG	acylated (active) ghrelin
Akt/AKT	serine/threonine-protein kinase or protein kinase B (PKB)
APC	adenomatous polyposis coli
BMI	body mass index
BRAF	a human gene that encodes protein called B-Raf
cAMP	*cyclic adenosine monophosphate*
CC	*colon cancer*
CI	confidence interval
CRC	colorectal cancer
EGFR	epidermal growth factor receptor
ERK	extracellular signal-regulated kinase
GH	growth hormone
GHRLOS	the ghrelin opposite strand/antisense non-coding RNA
GLP-1	glucagon-like peptide-1
GOAT	ghrelin-O-acyl-transferase
HOMA-IR	homeostatic model assessment-insulin resistance
HR	hazard ratio
IGF-1, -2	insulin-like growth factor 1, -2
IGFBPs	IGF binding proteins
IR	insulin resistance
KRAS	oncogene found in Kirsten rat sarcoma virus
LBM	lean body mass
mTOR	the mammalian target of rapamycin; protein kinase from PI3K family
OD	odds ratio
PI3K	phosphoinositide 3-kinase
PTEN	phosphatase and tensin homolog deleted on chromosome ten
Ras	“Rat sarcoma virus” protein, small GTP-ase
RC	rectal cancer
SNPs	single nucleotide polymorphisms
TNF-α	tumour necrosis factor alpha
UC	ulcerative colitis
UnAG	des/unacylated ghrelin

## References

[1] Gahete M.D., Córdoba-Chacón J., Hergueta-Redondo M., Martínez-Fuentes A.J., Kineman R.D., Moreno-Bueno G., Luque R.M., Castaño J.P. (2011). A novel human ghrelin variant (In1-ghrelin) and ghrelin-O-acyltransferase are overexpressed in breast cancer: Potential pathophysiological relevance. PLoS ONE.

[2] Gahete M.D., Rincón-Fernández D., Villa-Osaba A., Hormaechea-Agulla D., Ibáñez-Costa A., Martínez-Fuentes A.J., Gracia-Navarro F., Castaño J.P., Luque R.M. (2013). Ghrelin gene products, receptors, and GOAT enzyme: Biological and pathophysiological insight. J. Endocrinol..

[3] Delhanty P.J., Neggers S.J., van der Lely A.J. (2014). Should we consider des-acyl ghrelin as a separate hormone and if so, what does it do?. Front. Horm. Res..

[4] Luque R.M., Sampedro-Nuñez M., Gahete M.D., Ramos-Levi A., Ibáñez-Costa A., Rivero-Cortés E., Serrano-Somavilla A., Adrados M., Culler M.D., Castaño J.P. (2015). In1-ghrelin, a splice variant of ghrelin gene, is associated with the evolution and aggressiveness of human neuroendocrine tumors: Evidence from clinical, cellular and molecular parameters. Oncotarget.

[5] Lv Y., Liang T., Wang G., Li Z. (2018). Ghrelin, a gastrointestinal hormone, regulates energy balance and lipid metabolism. Biosci. Rep..

[6] Soleyman-Jahi S., Sadeghi F., Khoshbin A.P., Khani L., Roosta V., Zendehdel K. (2019). Attribution of Ghrelin to Cancer; Attempts to Unravel an Apparent Controversy. Front. Oncol..

[7] Davis T.R., Pierce M.R., Novak S.X., Hougland J.L. (2021). Ghrelin octanoylation by ghrelin *O*-acyltransferase: Protein acylation impacting metabolic and neuroendocrine signalling. Open Biol..

[8] Seim I., Collet C., Herington A.C., Chopin L.K. (2007). Revised genomic structure of the human ghrelin gene and identification of novel exons, alternative splice variants and natural antisense transcripts. BMC Genom..

[9] Cappellari G.G., Barazzoni R. (2019). Ghrelin forms in the modulation of energy balance and metabolism. Eat. Weight Disord. Stud. Anorex. Bulim. Obes..

[10] Kojima M., Hosoda H., Date Y., Nakazato M., Matsuo H., Kangawa K. (1999). Ghrelin is a growth-hormone-releasing acylated peptide from stomach. Nature.

[11] Kojima M., Hosoda H., Kangawa K. (2012). Purification of rat and human ghrelins. Methods Enzymol..

[12] Soares J.B., Leite-Moreira A.F. (2008). Ghrelin, des-acyl ghrelin and obestatin: Three pieces of the same puzzle. Peptides.

[13] Seim I., Amorim L., Walpole C., Carter S., Chopin L.K., Herington A.C. (2010). Ghrelin gene-related peptides: Multifunctional endocrine/autocrine modulators in health and disease. Clin. Exp. Pharmacol. Physiol..

[14] Müller T.D., Nogueiras R., Andermann M.L., Andrews Z.B., Anker S.D., Argente J., Batterham R.L., Benoit S.C., Bowers C.Y., Broglio F. (2015). Ghrelin. Mol. Metab..

[15] Akalu Y., Molla M.D., Dessie G., Ayelign B. (2020). Physiological Effect of Ghrelin on Body Systems. Int. J. Endocrinol..

[16] Schalla M.A., Taché Y., Stengel A. (2021). Neuroendocrine Peptides of the Gut and Their Role in the Regulation of Food Intake. Compr. Physiol..

[17] Andrews Z.B. (2019). Ghrelin: What’s the function?. J. Neuroendocrinol..

[18] Howard A.D., Feighner S.D., Cully D.F., Arena J.P., Liberator P.A., Rosenblum C.I., Hamelin M., Hreniuk D.L., Palyha O.C., Anderson J. (1996). A receptor in pituitary and hypothalamus that functions in growth hormone release. Science.

[19] de la Cour C.D., Björkqvist M., Sandvik A.K., Bakke I., Zhao C.M., Chen D., Håkanson R. (2001). A-like cells in the rat stomach contain ghrelin and do not operate under gastrin control. Regul. Pept..

[20] Davenport A.P., Bonner T.I., Foord S.M., Harmar A.J., Neubig R.R., Pin J.P., Spedding M., Kojima M., Kangawa K. (2005). International Union of Pharmacology. LVI. Ghrelin receptor nomenclature, distribution, and function. Pharmacol. Rev..

[21] Sato T., Nakamura Y., Shiimura Y., Ohgusu H., Kangawa K., Kojima M. (2012). Structure, regulation and function of ghrelin. J. Biochem..

[22] Camiña J.P. (2006). Cell biology of the ghrelin receptor. J. Neuroendocrinol..

[23] McKee K.K., Palyha O.C., Feighner S.D., Hreniuk D.L., Tan C.P., Phillips M.S., Smith R.G., Van der Ploeg L.H., Howard A.D. (1997). Molecular analysis of rat pituitary and hypothalamic growth hormone secretagogue receptors. Mol. Endocrinol..

[24] Petersenn S., Rasch A.C., Penshorn M., Beil F.U., Schulte H.M. (2001). Genomic structure and transcriptional regulation of the human growth hormone secretagogue receptor. Endocrinology.

[25] van der Lely A.J., Tschöp M., Heiman M.L., Ghigo E. (2004). Biological, physiological, pathophysiological, and pharmacological aspects of ghrelin. Endocr. Rev..

[26] Tian P., Lu X., Jin N., Shi J. (2020). Knockdown of ghrelin-O-acyltransferase attenuates colitis through the modulation of inflammatory factors and tight junction proteins in the intestinal epithelium. Cell Biol. Int..

[27] Bednarek M.A., Feighner S.D., Pong S.S., McKee K.K., Hreniuk D.L., Silva M.V., Warren V.A., Howard A.D., Van Der Ploeg L.H., Heck J.V. (2000). Structure-function studies on the new growth hormone-releasing peptide, ghrelin: Minimal sequence of ghrelin necessary for activation of growth hormone secretagogue receptor 1a. J. Med. Chem..

[28] Matsumoto M., Hosoda H., Kitajima Y., Morozumi N., Minamitake Y., Tanaka S., Matsuo H., Kojima M., Hayashi Y., Kangawa K. (2001). Structure-activity relationship of ghrelin: Pharmacological study of ghrelin peptides. Biochem. Biophys. Res. Commun..

[29] Banks W.A., Tschöp M., Robinson S.M., Heiman M.L. (2002). Extent and direction of ghrelin transport across the blood-brain barrier is determined by its unique primary structure. J. Pharmacol. Exp. Ther..

[30] Germain N., Cuenco J., Ling Y., Minnion J.S., Bageacu S., Grouselle D., Estour B., Galusca B. (2019). Ghrelin acylation by ghrelin-O-acyltransferase can occur in healthy part of oncological liver in humans. Am. J. Physiol. Gastrointest. Liver Physiol..

[31] Ariyasu H., Takaya K., Tagami T., Ogawa Y., Hosoda K., Akamizu T., Suda M., Koh T., Natsui K., Toyooka S. (2001). Stomach is a major source of circulating ghrelin, and feeding state determines plasma ghrelin-like immunoreactivity levels in humans. J. Clin. Endocrinol. Metab..

[32] Ueberberg B., Unger N., Saeger W., Mann K., Petersenn S. (2009). Expression of ghrelin and its receptor in human tissues. Horm. Metab. Res..

[33] Stengel A., Goebel M., Wang L., Taché Y. (2010). Ghrelin, des-acyl ghrelin and nesfatin-1 in gastric X/A-like cells: Role as regulators of food intake and body weight. Peptides.

[34] Kerbel B., Unniappan S. (2012). Nesfatin-1 suppresses energy intake, co-localises ghrelin in the brain and gut, and alters ghrelin, cholecystokinin and orexin mRNA expression in goldfish. J. Neuroendocrinol..

[35] LeSauter J., Hoque N., Weintraub M., Pfaff D.W., Silver R. (2009). Stomach ghrelin-secreting cells as food-entrainable circadian clocks. Proc. Natl. Acad. Sci. USA.

[36] Shiiya T., Nakazato M., Mizuta M., Date Y., Mondal M.S., Tanaka M., Nozoe S., Hosoda H., Kangawa K., Matsukura S. (2002). Plasma ghrelin levels in lean and obese humans and the effect of glucose on ghrelin secretion. J. Clin. Endocrinol. Metab..

[37] Sun Y., Wang P., Zheng H., Smith R.G. (2004). Ghrelin stimulation of growth hormone release and appetite is mediated through the growth hormone secretagogue receptor. Proc. Natl. Acad. Sci. USA.

[38] Spiegel K., Tasali E., Leproult R., Scherberg N., Van Cauter E. (2011). Twenty-four-hour profiles of acylated and total ghrelin: Relationship with glucose levels and impact of time of day and sleep. J. Clin. Endocrinol. Metab..

[39] Ertosun M.G., Kocak G., Ozes O.N. (2019). The regulation of circadian clock by tumor necrosis factor alpha. Cytokine Growth Factor Rev..

[40] Konturek P.C., Brzozowski T., Konturek S.J. (2011). Gut clock: Implication of circadian rhythms in the gastrointestinal tract. J. Physiol. Pharmacol..

[41] Masuda Y., Tanaka T., Inomata N., Ohnuma N., Tanaka S., Itoh Z., Hosoda H., Kojima M., Kangawa K. (2000). Ghrelin stimulates gastric acid secretion and motility in rats. Biochem. Biophys. Res. Commun..

[42] Sallam H.S., Chen J.D. (2010). The prokinetic face of ghrelin. Int. J. Pept..

[43] Sanger G.J., Broad J., Callaghan B., Furness J.B. (2017). Ghrelin and Motilin Control Systems in GI Physiology and Therapeutics. Handb. Exp. Pharmacol..

[44] Noh J.Y., Wu C.S., DeLuca J.A.A., Devaraj S., Jayaraman A., Alaniz R.C., Tan X.D., Allred C.D., Sun Y. (2022). Novel Role of Ghrelin Receptor in Gut Dysbiosis and Experimental Colitis in Aging. Int. J. Mol. Sci..

[45] Koutouratsas T., Kalli T., Karamanolis G., Gazouli M. (2019). Contribution of ghrelin to functional gastrointestinal disorders’ pathogenesis. World J. Gastroenterol..

[46] Yamada C. (2021). Involvement of Ghrelin Dynamics in Stress-Induced Eating Disorder: Effects of Sex and Aging. Int. J. Mol. Sci..

[47] El-Salhy M., Solomon T., Hausken T., Gilja O.H., Hatlebakk J.G. (2017). Gastrointestinal neuroendocrine peptides/amines in inflammatory bowel disease. World J. Gastroenterol..

[48] El-Salhy M. (2009). Ghrelin in gastrointestinal diseases and disorders: A possible role in the pathophysiology and clinical implications (review). Int. J. Mol. Med..

[49] Mathur N., Mehdi S.F., Anipindi M., Aziz M., Khan S.A., Kondakindi H., Lowell B., Wang P., Roth J. (2021). Ghrelin as an Anti-Sepsis Peptide: Review. Front. Immunol..

[50] Karaskova E., Velganova-Veghova M., Geryk M., Foltenova H., Kucerova V., Karasek D. (2021). Role of Adipose Tissue in Inflammatory Bowel Disease. Int. J. Mol. Sci..

[51] Lin T.C., Hsiao M. (2017). Ghrelin and cancer progression. Biochim. Biophys. Acta Rev. Cancer..

[52] Papotti M., Cassoni P., Volante M., Deghenghi R., Muccioli G., Ghigo E. (2001). Ghrelin-producing endocrine tumors of the stomach and intestine. J. Clin. Endocrinol. Metab..

[53] Ekeblad S., Lejonklou M.H., Grimfjärd P., Johansson T., Eriksson B., Grimelius L., Stridsberg M., Stålberg P., Skogseid B. (2007). Co-expression of ghrelin and its receptor in pancreatic endocrine tumours. Clin. Endocrinol..

[54] Herrera-Martínez A.D., Gahete M.D., Sánchez-Sánchez R., Alors-Perez E., Pedraza-Arevalo S., Serrano-Blanch R., Martínez-Fuentes A.J., Gálvez-Moreno M.A., Castaño J.P., Luque R.M. (2018). Ghrelin-O-Acyltransferase (GOAT) Enzyme as a Novel Potential Biomarker in Gastroenteropancreatic Neuroendocrine Tumors. Clin. Transl. Gastroenterol..

[55] Zhu C.Z., Liu D., Kang W.M., Yu J.C., Ma Z.Q., Ye X., Li K. (2017). Ghrelin and gastrointestinal stromal tumors. World J. Gastroenterol..

[56] Spiridon I.A., Ciobanu D.G.A., Giușcă S.E., Ferariu D., Pleşca I.C., Căruntu I.D. (2021). GIST and Ghrelin: To Be or Not to Be?. Diagnostics.

[57] Sever S., White D.L., Garcia J.M. (2016). Is there an effect of ghrelin/ghrelin analogs on cancer? A systematic review. Endocr. Relat. Cancer.

[58] Spiridon I.A., Ciobanu D.G.A., Giușcă S.E., Căruntu I.D. (2021). Ghrelin and its role in gastrointestinal tract tumors (Review). Mol. Med. Rep..

[59] Waseem T., Rehman J.U., Ahmad F., Azam M., Qureshi M.A. (2008). Role of ghrelin axis in colorectal cancer: A novel association. Peptides.

[60] Waseem T., Duxbury M., Ashley S.W., Robinson M.K. (2014). Ghrelin promotes intestinal epithelial cell proliferation through PI3K/Akt pathway and EGFR trans-activation both converging to ERK 1/2 phosphorylation. Peptides.

[61] Liu A., Huang C., Xu J., Cai X. (2016). Lentivirus-mediated shRNA interference of ghrelin receptor blocks proliferation in the colorectal cancer cells. Cancer Med..

[62] Lien G.S., Lin C.H., Yang Y.L., Wu M.S., Chen B.C. (2016). Ghrelin induces colon cancer cell proliferation through the GHS-R, Ras, PI3K, Akt, and mTOR signalling pathways. Eur. J. Pharmacol..

[63] Harada S., Morlote D. (2020). Molecular Pathology of Colorectal Cancer. Adv. Anat. Pathol..

[64] Sung H., Ferlay J., Siegel R.L., Laversanne M., Soerjomataram I., Jemal A., Bray F. (2021). Global cancer statistics 2020: GLOBOCAN estimates of incidence and mortality worldwide for 36 cancers in 185 countries. CA Cancer J. Clin..

[65] Huang D., Sun W., Zhou Y., Li P., Chen F., Chen H., Xia D., Xu E., Lai M., Wu Y. (2018). Mutations of key driver genes in colorectal cancer progression and metastasis. Cancer Metastasis Rev..

[66] Yang Z.H., Dang Y.Q., Ji G. (2019). Role of epigenetics in transformation of inflammation into colorectal cancer. World J. Gastroenterol..

[67] Uyar G.O., Sanlier N. (2019). Association of Adipokines and Insulin, Which Have a Role in Obesity, with Colorectal Cancer. Eurasian J. Med..

[68] Biller L.H., Schrag D. (2021). Diagnosis and Treatment of Metastatic Colorectal Cancer: A Review. JAMA.

[69] Schmitt M., Greten F.R. (2021). The inflammatory pathogenesis of colorectal cancer. Nat. Rev. Immunol..

[70] Harlid S., Harbs J., Myte R., Brunius C., Gunter M.J., Palmqvist R., Liu X., Van Guelpen B. (2021). A two-tiered targeted proteomics approach to identify pre-diagnostic biomarkers of colorectal cancer risk. Sci. Rep..

[71] Harlid S., Gunter M.J., Van Guelpen B. (2021). Risk-Predictive and Diagnostic Biomarkers for Colorectal Cancer; a Systematic Review of Studies Using Pre-Diagnostic Blood Samples Collected in Prospective Cohorts and Screening Settings. Cancers.

[72] Nikolopoulos D., Theocharis S., Kouraklis G. (2010). Ghrelin’s role on gastrointestinal tract cancer. Surg. Oncol..

[73] Chopin L., Walpole C., Seim I., Cunningham P., Murray R., Whiteside E., Josh P., Herington A. (2011). Ghrelin and cancer. Mol. Cell Endocrinol..

[74] Chopin L.K., Seim I., Walpole C.M., Herington A.C. (2012). The ghrelin axis--does it have an appetite for cancer progression?. Endocr. Rev..

[75] Date Y., Kojima M., Hosoda H., Sawaguchi A., Mondal M.S., Suganuma T., Matsukura S., Kangawa K., Nakazato M. (2000). Ghrelin, a novel growth hormone-releasing acylated peptide, is synthesized in a distinct endocrine cell type in the gastrointestinal tracts of rats and humans. Endocrinology.

[76] Sakata I., Sakai T. (2010). Ghrelin cells in the gastrointestinal tract. Int. J. Pept..

[77] Hosoda H., Kojima M., Matsuo H., Kangawa K. (2000). Purification and characterization of rat des-Gln14-Ghrelin, a second endogenous ligand for the growth hormone secretagogue receptor. J. Biol. Chem..

[78] Mondal M.S., Toshinai K., Ueno H., Koshinaka K., Nakazato M. (2008). Characterization of obestatin in rat and human stomach and plasma, and its lack of acute effect on feeding behavior in rodents. J. Endocrinol..

[79] Fothergill L.J., Furness J.B. (2018). Diversity of enteroendocrine cells investigated at cellular and subcellular levels: The need for a new classification scheme. Histochem. Cell Biol..

[80] Sakata I., Nakamura K., Yamazaki M., Matsubara M., Hayashi Y., Kangawa K., Sakai T. (2002). Ghrelin-producing cells exist as two types of cells, closed- and opened-type cells, in the rat gastrointestinal tract. Peptides.

[81] Zhao Z., Sakai T. (2008). Characteristic features of ghrelin cells in the gastrointestinal tract and the regulation of stomach ghrelin expression and production. World J. Gastroenterol..

[82] Egerod K.L., Engelstoft M.S., Grunddal K.V., Nøhr M.K., Secher A., Sakata I., Pedersen J., Windeløv J.A., Füchtbauer E.M., Olsen J. (2012). A major lineage of enteroendocrine cells coexpress CCK, secretin, GIP, GLP-1, PYY, and neurotensin but not somatostatin. Endocrinology.

[83] Fothergill L.J., Callaghan B., Hunne B., Bravo D.M., Furness J.B. (2017). Costorage of Enteroendocrine Hormones Evaluated at the Cell and Subcellular Levels in Male Mice. Endocrinology.

[84] Martins P., Fakhry J., de Oliveira E.C., Hunne B., Fothergill L.J., Ringuet M., Reis D.D., Rehfeld J.F., Callaghan B., Furness J.B. (2017). Analysis of enteroendocrine cell populations in the human colon. Cell Tissue Res..

[85] Volante M., Allìa E., Gugliotta P., Funaro A., Broglio F., Deghenghi R., Muccioli G., Ghigo E., Papotti M. (2002). Expression of ghrelin and of the GH secretagogue receptor by pancreatic islet cells and related endocrine tumors. J. Clin. Endocrinol. Metab..

[86] Elabadlah H., Hameed R., D’Souza C., Mohsin S., Adeghate E.A. (2020). Exogenous Ghrelin Increases Plasma Insulin Level in Diabetic Rats. Biomolecules.

[87] Date Y., Nakazato M., Hashiguchi S., Dezaki K., Mondal M.S., Hosoda H., Kojima M., Kangawa K., Arima T., Matsuo H. (2002). Ghrelin is present in pancreatic alpha-cells of humans and rats and stimulates insulin secretion. Diabetes.

[88] Andralojc K.M., Mercalli A., Nowak K.W., Albarello L., Calcagno R., Luzi L., Bonifacio E., Doglioni C., Piemonti L. (2009). Ghrelin-producing epsilon cells in the developing and adult human pancreas. Diabetologia.

[89] Rindi G., Necchi V., Savio A., Torsello A., Zoli M., Locatelli V., Raimondo F., Cocchi D., Solcia E. (2002). Characterisation of gastric ghrelin cells in man and other mammals: Studies in adult and fetal tissues. Histochem. Cell Biol..

[90] Mitrović O., Mićić M., Radenković G., Vignjević S., Ðikić D., Budeč M., Breković T., Čokić V. (2012). Endocrine cells in human fetal corpus of stomach: Appearance, distribution, and density. J. Gastroenterol..

[91] Mitrović O., Čokić V., Đikić D., Budeč M., Vignjević S., Subotički T., Diklić M., Ajtić R. (2014). Ghrelin receptors in human gastrointestinal tract during prenatal and early postnatal development. Peptides.

[92] Papotti M., Ghè C., Cassoni P., Catapano F., Deghenghi R., Ghigo E., Muccioli G. (2000). Growth hormone secretagogue binding sites in peripheral human tissues. J. Clin. Endocrinol. Metab..

[93] Gnanapavan S., Kola B., Bustin S.A., Morris D.G., McGee P., Fairclough P., Bhattacharya S., Carpenter R., Grossman A.B., Korbonits M. (2002). The tissue distribution of the mRNA of ghrelin and subtypes of its receptor, GHS-R, in humans. J. Clin. Endocrinol. Metab..

[94] Dass N.B., Munonyara M., Bassil A.K., Hervieu G.J., Osbourne S., Corcoran S., Morgan M., Sanger G.J. (2003). Growth hormone secretagogue receptors in rat and human gastrointestinal tract and the effects of ghrelin. Neuroscience.

[95] Wang Z., Wang W., Qiu W., Fan Y., Zhao J., Wang Y., Zheng Q. (2007). Involvement of ghrelin-growth hormone secretagogue receptor system in pathoclinical profiles of digestive system cancer. Acta Biochim. Biophys. Sin..

[96] Stojsavljevic-Shapeski S., Virovic-Jukic L., Tomas D., Duvnjak M., Tomasic V., Hrabar D., Kralj D., Budimir I., Barsic N., Ljubicic N. (2021). Expression of adipokine ghrelin and ghrelin receptor in human colorectal adenoma and correlation with the grade of dysplasia. World J. Gastrointest. Surg..

[97] Lim C.T., Kola B., Grossman A., Korbonits M. (2011). The expression of ghrelin O-acyltransferase (GOAT) in human tissues. Endocr. J..

[98] Yang J., Brown M.S., Liang G., Grishin N.V., Goldstein J.L. (2008). Identification of the acyltransferase that octanoylates ghrelin, an appetite-stimulating peptide hormone. Cell.

[99] Sakata I., Yang J., Lee C.E., Osborne-Lawrence S., Rovinsky S.A., Elmquist J.K., Zigman J.M. (2009). Colocalization of ghrelin O-acyltransferase and ghrelin in gastric mucosal cells. Am. J. Physiol. Endocrinol. Metab..

[100] Goebel-Stengel M., Hofmann T., Elbelt U., Teuffel P., Ahnis A., Kobelt P., Lambrecht N.W., Klapp B.F., Stengel A. (2013). The ghrelin activating enzyme ghrelin-O-acyltransferase (GOAT) is present in human plasma and expressed dependent on body mass index. Peptides.

[101] Dezaki K., Yada T. (2022). Status of ghrelin as an islet hormone and paracrine/autocrine regulator of insulin secretion. Peptides.

[102] Date Y., Nakazato M., Murakami N., Kojima M., Kangawa K., Matsukura S. (2001). Ghrelin acts in the central nervous system to stimulate gastric acid secretion. Biochem. Biophys. Res. Commun..

[103] Sakurada T., Ro S., Onouchi T., Ohno S., Aoyama T., Chinen K., Takabayashi H., Kato S., Takayama K., Yakabi K. (2010). Comparison of the actions of acylated and desacylated ghrelin on acid secretion in the rat stomach. J. Gastroenterol..

[104] Gupta D., Patterson A.M., Osborne-Lawrence S., Bookout A.L., Varshney S., Shankar K., Singh O., Metzger N.P., Richard C.P., Wyler S.C. (2021). Disrupting the ghrelin-growth hormone axis limits ghrelin’s orexigenic but not glucoregulatory actions. Mol. Metab..

[105] Verbeure W., van Goor H., Mori H., van Beek A.P., Tack J., van Dijk P.R. (2021). The Role of Gasotransmitters in Gut Peptide Actions. Front. Pharmacol..

[106] Asakawa A., Inui A., Kaga T., Yuzuriha H., Nagata T., Ueno N., Makino S., Fujimiya M., Niijima A., Fujino M.A. (2001). Ghrelin is an appetite-stimulatory signal from stomach with structural resemblance to motilin. Gastroenterology.

[107] Tomasetto C., Wendling C., Rio M.C., Poitras P. (2001). Identification of cDNA encoding motilin related peptide/ghrelin precursor from dog fundus. Peptides.

[108] Broad J., Góralczyk A., Mannur K., Dukes G.E., Sanger G.J. (2014). Drugs acting at 5-HT4, D2, motilin, and ghrelin receptors differ markedly in how they affect neuromuscular functions in human isolated stomach. Neurogastroenterol. Motil..

[109] Deloose E., Vos R., Corsetti M., Depoortere I., Tack J. (2015). Endogenous motilin, but not ghrelin plasma levels fluctuate in accordance with gastric phase III activity of the migrating motor complex in man. Neurogastroenterol. Motil..

[110] Tack J., Depoortere I., Bisschops R., Delporte C., Coulie B., Meulemans A., Janssens J., Peeters T. (2006). Influence of ghrelin on interdigestive gastrointestinal motility in humans. Gut.

[111] Cornejo M.P., Denis R.G.P., Romero G.G., Fernández G., Reynaldo M., Luquet S., Perello M. (2021). Ghrelin treatment induces rapid and delayed increments of food intake: A heuristic model to explain ghrelin’s orexigenic effects. Cell Mol. Life Sci..

[112] Lee H.M., Wang G., Englander E.W., Kojima M., Greeley G.H. (2002). Ghrelin, a new gastrointestinal endocrine peptide that stimulates insulin secretion: Enteric distribution, ontogeny, influence of endocrine, and dietary manipulations. Endocrinology.

[113] Nawrot-Porabka K., Jaworek J., Leja-Szpak A., Szklarczyk J., Macko M., Kot M., Mitis-Musioł M., Konturek S.J., Pawlik W.W. (2007). The effect of luminal ghrelin on pancreatic enzyme secretion in the rat. Regul. Pept..

[114] Arosio M., Ronchi C.L., Gebbia C., Cappiello V., Beck-Peccoz P., Peracchi M. (2003). Stimulatory effects of ghrelin on circulating somatostatin and pancreatic polypeptide levels. J. Clin. Endocrinol. Metab..

[115] Broglio F., Arvat E., Benso A., Gottero C., Muccioli G., Papotti M., van der Lely A.J., Deghenghi R., Ghigo E. (2001). Ghrelin, a natural GH secretagogue produced by the stomach, induces hyperglycemia and reduces insulin secretion in humans. J. Clin. Endocrinol. Metab..

[116] Tong J., Davis H.W., Summer S., Benoit S.C., Haque A., Bidlingmaier M., Tschöp M.H., D’Alessio D. (2014). Acute administration of unacylated ghrelin has no effect on Basal or stimulated insulin secretion in healthy humans. Diabetes.

[117] Granata R., Volante M., Settanni F., Gauna C., Ghé C., Annunziata M., Deidda B., Gesmundo I., Abribat T., van der Lely A.J. (2010). Unacylated ghrelin and obestatin increase islet cell mass and prevent diabetes in streptozotocin-treated newborn rats. J. Mol. Endocrinol..

[118] Bassil A.K., Dass N.B., Murray C.D., Muir A., Sanger G.J. (2005). Prokineticin-2, motilin, ghrelin and metoclopramide: Prokinetic utility in mouse stomach and colon. Eur. J. Pharmacol..

[119] Tebbe J.J., Mronga S., Tebbe C.G., Ortmann E., Arnold R., Schäfer M.K. (2005). Ghrelin-induced stimulation of colonic propulsion is dependent on hypothalamic neuropeptide Y1- and corticotrophin-releasing factor 1 receptor activation. J. Neuroendocrinol..

[120] Shimizu Y., Chang E.C., Shafton A.D., Ferens D.M., Sanger G.J., Witherington J., Furness J.B. (2006). Evidence that stimulation of ghrelin receptors in the spinal cord initiates propulsive activity in the colon of the rat. J. Physiol..

[121] Hirayama H., Shiina T., Shima T., Kuramoto H., Takewaki T., Furness J.B., Shimizu Y. (2010). Contrasting effects of ghrelin and des-acyl ghrelin on the lumbo-sacral defecation center and regulation of colorectal motility in rats. Neurogastroenterol. Motil..

[122] Cummings D.E., Weigle D.S., Frayo R.S., Breen P.A., Ma M.K., Dellinger E.P., Purnell J.Q. (2002). Plasma ghrelin levels after diet-induced weight loss or gastric bypass surgery. N. Engl. J. Med..

[123] Foster-Schubert K.E., Overduin J., Prudom C.E., Liu J., Callahan H.S., Gaylinn B.D., Thorner M.O., Cummings D.E. (2008). Acyl and total ghrelin are suppressed strongly by ingested proteins, weakly by lipids, and biphasically by carbohydrates. J. Clin. Endocrinol. Metab..

[124] Tatsuguchi A., Miyake K., Gudis K., Futagami S., Tsukui T., Wada K., Kishida T., Fukuda Y., Sugisaki Y., Sakamoto C. (2004). Effect of *Helicobacter pylori* infection on ghrelin expression in human gastric mucosa. Am. J. Gastroenterol..

[125] Seim I., Carter S.L., Herington A.C., Chopin L.K. (2008). Complex organisation and structure of the ghrelin antisense strand gene GHRLOS, a candidate non-coding RNA gene. BMC Mol. Biol..

[126] Hosoda H., Kangawa K. (2008). The autonomic nervous system regulates gastric ghrelin secretion in rats. Regul. Pept..

[127] De la Cour C.D., Norlén P., Håkanson R. (2007). Secretion of ghrelin from rat stomach ghrelin cells in response to local microinfusion of candidate messenger compounds: A microdialysis study. Regul. Pept..

[128] Rodríguez A. (2014). Novel molecular aspects of ghrelin and leptin in the control of adipobiology and the cardiovascular system. Obes. Facts.

[129] Freeman H.J. (2004). Risk of gastrointestinal malignancies and mechanisms of cancer development with obesity and its treatment. Best Pract. Res. Clin. Gastroenterol..

[130] Riondino S., Roselli M., Palmirotta R., Della-Morte D., Ferroni P., Guadagni F. (2014). Obesity and colorectal cancer: Role of adipokines in tumor initiation and progression. World J. Gastroenterol..

[131] Joshi R.K., Kim W.J., Lee S.A. (2014). Association between obesity-related adipokines and colorectal cancer: A case-control study and meta-analysis. World J. Gastroenterol..

[132] Campa D., Pardini B., Naccarati A., Vodickova L., Novotny J., Steinke V., Rahner N., Holinski-Feder E., Morak M., Schackert H.K. (2010). Polymorphisms of genes coding for ghrelin and its receptor in relation to colorectal cancer risk: A two-step gene-wide case-control study. BMC Gastroenterol..

[133] Mahmoudi T., Karimi K., Arkani M., Farahani H., Vahedi M., Dabiri R., Nobakht H., Asadi A., Mirakhorli M., Arshi B. (2014). Resistin -420C>G promoter variant and colorectal cancer risk. Int. J. Biol. Markers.

[134] Pabalan N.A., Seim I., Jarjanazi H., Chopin L.K. (2014). Associations between ghrelin and ghrelin receptor polymorphisms and cancer in Caucasian populations: A meta-analysis. BMC Genet..

[135] Zhu S., Shao B., Hao Y., Li Z., Liu H., Li H., Wang M., Wang K. (2015). No association of single nucleotide polymorphisms involved in GHRL and GHSR with cancer risk: A meta-analysis. Cancer Biomark..

[136] Coppedè F., Stoccoro A., Lazzarotti A., Spisni R., Migliore L. (2018). Investigation of GHSR and GHRL methylation in colorectal cancer. Epigenomics.

[137] D’Onghia V., Leoncini R., Carli R., Santoro A., Giglioni S., Sorbellini F., Marzocca G., Bernini A., Campagna S., Marinello E. (2007). Circulating gastrin and ghrelin levels in patients with colorectal cancer: Correlation with tumour stage, *Helicobacter pylori* infection and BMI. Biomed. Pharmacother..

[138] Kemik O., Sumer A., Kemik A.S., Hasirci I., Purisa S., Dulger A.C., Demiriz B., Tuzun S. (2010). The relationship among acute-phase response proteins, cytokines and hormones in cachectic patients with colon cancer. World J. Surg. Oncol..

[139] Zygulska A.L., Furgala A., Krzemieniecki K., Kaszuba-Zwoinska J., Thor P. (2017). Enterohormonal disturbances in colorectal cancer patients. Neoplasma.

[140] Asadi A., Farahani H., Mahmoudi T., Tabaeian S.P., Rezamand G., Mohammadbeigi A., Dabiri R., Nobakht H., Rezvan S., Mohammadi F. (2021). Circulating ghrelin levels and susceptibility to colorectal câncer. Arq. Gastroenterol..

[141] Kosova F., Coskun T., Kaya Y., Kara E., Ari Z. (2013). Adipocytokine levels of colon cancer patients before and after treatment. Bratisl. Lek. Listy..

[142] Murphy G., Cross A.J., Dawsey S.M., Stanczyk F.Z., Kamangar F., Weinstein S.J., Taylor P.R., Männistö S., Albanes D., Abnet C.C. (2018). Serum ghrelin is associated with risk of colorectal adenocarcinomas in the ATBC study. Gut.

[143] Nikolopoulos D., Theocharis S., Moutsios-Rentzos A., Kouraklis G., Kostakis A. (2014). The role of serum total ghrelin level elevation in colon cancer patients. J. BUON.

[144] Zhu C., Liu Y., Kang W., Zhang Z., Zeng Z., Liu D. (2020). Exploration of the role of serum ghrelin in the diagnosis and treatment of digestive tract malignancies. J. Int. Med. Res..

[145] Wolf I., Sadetzki S., Kanety H., Kundel Y., Pariente C., Epstein N., Oberman B., Catane R., Kaufman B., Shimon I. (2006). Adiponectin, ghrelin, and leptin in cancer cachexia in breast and colon cancer patients. Cancer.

[146] Huang Q., Fan Y.Z., Ge B.J., Zhu Q., Tu Z.Y. (2007). Circulating ghrelin in patients with gastric or colorectal cancer. Dig. Dis. Sci..

[147] Shinsyu A., Bamba S., Kurihara M., Matsumoto H., Sonoda A., Inatomi O., Andoh A., Takebayashi K., Kojima M., Iida H. (2020). Inflammatory cytokines, appetite-regulating hormones, and energy metabolism in patients with gastrointestinal cancer. Oncol. Lett..

[148] Sundkvist A., Myte R., Palmqvist R., Harlid S., Van Guelpen B. (2019). Plasma ghrelin is probably not a useful biomarker for risk prediction or early detection of colorectal cancer. Gut.

[149] Wu S., Liu J., Wang X., Li M., Chen Z., Tang Y. (2017). Aberrant Expression of the Long Non-coding RNA *GHRLOS* and Its Prognostic Significance in Patients with Colorectal Cancer. J. Cancer..

[150] He X.T., Fan X.M., Zha X.L. (2011). Ghrelin inhibits 5-fluorouracil-induced apoptosis in colonic cancer cells. J. Gastroenterol. Hepatol..

[151] Bułdak R.J., Pilc-Gumuła K., Bułdak Ł., Witkowska D., Kukla M., Polaniak R., Zwirska-Korczala K. (2015). Effects of ghrelin, leptin and melatonin on the levels of reactive oxygen species, antioxidant enzyme activity and viability of the HCT 116 human colorectal carcinoma cell line. Mol. Med. Rep..

[152] Bonfili L., Cuccioloni M., Cecarini V., Mozzicafreddo M., Palermo F.A., Cocci P., Angeletti M., Eleuteri A.M. (2013). Ghrelin induces apoptosis in colon adenocarcinoma cells via proteasome inhibition and autophagy induction. Apoptosis.

[153] Murata M., Okimura Y., Iida K., Matsumoto M., Sowa H., Kaji H., Kojima M., Kangawa K., Chihara K. (2002). Ghrelin modulates the downstream molecules of insulin signalling in hepatoma cells. J. Biol. Chem..

[154] Duxbury M.S., Waseem T., Ito H., Robinson M.K., Zinner M.J., Ashley S.W., Whang E.E. (2003). Ghrelin promotes pancreatic adenocarcinoma cellular proliferation and invasiveness. Biochem. Biophys. Res. Commun..

[155] Tian C., Zhang L., Hu D., Ji J. (2013). Ghrelin induces gastric cancer cell proliferation, migration, and invasion through GHS-R/NF-κB signalling pathway. Mol. Cell Biochem..

[156] Lawnicka H., Mełeń-Mucha G., Motylewska E., Mucha S., Stępień H. (2012). Modulation of ghrelin axis influences the growth of colonic and prostatic cancer cells in vitro. Pharmacol. Rep..

[157] Kawaguchi M., Kanemaru A., Fukushima T., Yamamoto K., Tanaka H., Haruyama Y., Itoh H., Matsumoto N., Kangawa K., Nakazato M. (2015). Ghrelin administration suppresses inflammation-associated colorectal carcinogenesis in mice. Cancer Sci..

[158] Peterson S.J., Mozer M. (2017). Differentiating Sarcopenia and Cachexia Among Patients With Cancer. Nutr. Clin. Pract..

[159] Zeng X., Chen S., Lin Y., Ke Z. (2018). Acylated and unacylated ghrelin inhibit apoptosis in myoblasts cocultured with colon carcinoma cells. Oncol. Rep..

[160] Daily J.W., Park S. (2022). Sarcopenia is a Cause and Consequence of Metabolic Dysregulation in Aging Humans: Effects of Gut Dysbiosis, Glucose Dysregulation, Diet and Lifestyle. Cells.

[161] Martin L., Muscaritoli M., Bourdel-Marchasson I., Kubrak C., Laird B., Gagnon B., Chasen M., Gioulbasanis I., Wallengren O., Voss A.C. (2021). Diagnostic criteria for cancer cachexia: Reduced food intake and inflammation predict weight loss and survival in an international, multi-cohort analysis. J. Cachexia Sarcopenia Muscle.

[162] Perboni S., Bowers C., Kojima S., Asakawa A., Inui A. (2008). Growth hormone releasing peptide 2 reverses anorexia associated with chemotherapy with 5-fluoruracil in colon cancer cell-bearing mice. World J. Gastroenterol..

[163] Villars F.O., Pietra C., Giuliano C., Lutz T.A., Riediger T. (2017). Oral Treatment with the Ghrelin Receptor Agonist HM01 Attenuates Cachexia in Mice Bearing Colon-26 (C26) Tumors. Int. J. Mol. Sci..

[164] Zeng X., Chen P., Zhao L., Chen S. (2020). Acylated and unacylated ghrelin relieve cancer cachexia in mice through multiple mechanisms. Chin. J. Physiol..

[165] DeBoer M.D. (2012). The use of ghrelin and ghrelin receptor agonists as a treatment for animal models of disease: Efficacy and mechanism. Curr. Pharm. Des..

[166] Hamauchi S., Furuse J., Takano T., Munemoto Y., Furuya K., Baba H., Takeuchi M., Choda Y., Higashiguchi T., Naito T. (2019). A multicenter, open-label, single-arm study of anamorelin (ONO-7643) in advanced gastrointestinal cancer patients with cancer cachexia. Cancer.

[167] Lundholm K., Gunnebo L., Körner U., Iresjö B.M., Engström C., Hyltander A., Smedh U., Bosaeus I. (2010). Effects by daily long term provision of ghrelin to unselected weight-losing cancer patients: A randomized double-blind study. Cancer.

[168] Blum D., de Wolf-Linder S., Oberholzer R., Brändle M., Hundsberger T., Strasser F. (2021). Natural ghrelin in advanced cancer patients with cachexia, a case series. J. Cachexia Sarcopenia Muscle.

[169] Garcia J.M., Boccia R.V., Graham C.D., Yan Y., Duus E.M., Allen S., Friend J. (2015). Anamorelin for patients with cancer cachexia: An integrated analysis of two phase 2, randomised, placebo-controlled, double-blind trials. Lancet Oncol..

[170] Wakabayashi H., Arai H., Inui A. (2021). The regulatory approval of anamorelin for treatment of cachexia in patients with non-small cell lung cancer, gastric cancer, pancreatic cancer, and colorectal cancer in Japan: Facts and numbers. J. Cachexia Sarcopenia Muscle.

[171] Nakanishi Y., Higuchi J., Honda N., Komura N. (2021). Pharmacological profile and clinical efficacy of anamorelin HCl (ADLUMIZ^®^Tablets), the first orally available drug for cancer cachexia with ghrelin-like action in Japan. Nihon Yakurigaku Zasshi Folia Pharmacol. Jpn..

[172] Vergara-Fernandez O., Trejo-Avila M., Salgado-Nesme N. (2020). Sarcopenia in patients with colorectal cancer: A comprehensive review. World J. Clin. Cases.

[173] Garcia J.M., Polvino W.J. (2009). Pharmacodynamic hormonal effects of anamorelin, a novel oral ghrelin mimetic and growth hormone secretagogue in healthy volunteers. Growth Horm. IGF Res..

[174] Huang D.D., Wang S.L., Zhuang C.L., Zheng B.S., Lu J.X., Chen F.F., Zhou C.J., Shen X., Yu Z. (2015). Sarcopenia, as defined by low muscle mass, strength and physical performance, predicts complications after surgery for colorectal cancer. Colorectal Dis..

[175] Liu H., Sun D., Myasnikov A., Damian M., Baneres J.L., Sun J., Zhang C. (2021). Structural basis of human ghrelin receptor signalling by ghrelin and the synthetic agonist ibutamoren. Nat. Commun..

